# Accumulation differences of high-value ingredients in different phenotype *Lonicera macranthoides:* insights from integrative metabolome and transcriptome analyses

**DOI:** 10.3389/fpls.2025.1533263

**Published:** 2025-03-04

**Authors:** Juan Zeng, Yu Qing Long, Jia Yuan Zhu, Xue Sen Fu, Jing Yu Zhang, Jia Wei He, Xiao Rong Liu, Zhi Hui Wang, Qiao Zhen Tong, Xiang Dan Liu, Ri Bao Zhou

**Affiliations:** ^1^ School of Pharmacy, Hunan University of Chinese Medicine, Changsha, China; ^2^ Key Laboratory of Germplasm Resources and Standardized Planting of Hunan Large-scale Genuine Medicinal Materials, Changsha, China; ^3^ Department of Pharmacy, Yiyang Medical College, Yiyang, China; ^4^ Key Laboratory of Modern Research of TCM, Education Department of Hunan Province, Changsha, China

**Keywords:** *Lonicera macranthoides* Hand.-Mazz., metabolome, transcriptome, active ingredients, regulatory network

## Abstract

**Background:**

*Lonicera macranthoides* Hand.-Mazz., the primary sources of Lonicerae Flos(Shanyinhua), brings great medicinal and economic value as an invaluable source of natural bioactive compounds. Nutrient and metabolites accumulation generally changed accompany with its floral development and opening. While the specific accumulation pattern and the underlying molecular regulatory networks remain unclear.

**Methods:**

The present study intergrated a comparative analysis upon UPLC-MS/MS-based metabolomics and RNA-seq-based transcriptomics to revealed the differences in accumulation of flavonoids, phenolic acids, and terpenoids between the xianglei-type (corolla-closed) and wild-type (corolla-unfolded) of *L. macranthoides* flowers.

**Results and conclusion:**

674 differentially accumulated metabolites(DAMs) were identified in WT and XL, with 5,776 common differentially expressed genes(DEGs), revealing a significant differences in accumulation of flavonoids, phenolic acids, and terpenoids during the late stage of flower development between the xianglei-type and wild-type of *L. macranthoides* flowers. Combined analysis further identified 36 hub genes, major transcription factors and hormone-related genes, which play key roles in the differential accumulation of the abovementioned metabolites. These lines of evidences provide a molecular basis for the metabolic changes occurring during growth and can be significantly implicated in further research on the biosynthetic pathways associated with high-value potent active components in woody plants.

## 1 Introduction

*Lonicera macranthoides* Hand.-Mazz.(*LM*) presents a mainstream source of the TCM (traditional Chinese medicine), Lonicerae Flos *(shanyinhua)*, with various pharmacological effects such as promoting immunity, reducing blood levels of sugar and lipids, and anti-atherosclerosis effect (Chinese Pharmacopoeia Commission, C.P, 2005; Chen et al., 2015; Ma et al., 2022). According to the Yaozhi database(https://www.yaozh.com/), Lonicerae Flos flower extracts have been added to more than 100 Chinese patent medicine prescriptions as their main ingredients, which included cold cough tablets, stomatitis clear tablets, cold cough capsules, clear liver and gallbladder granules, psoriasis granules, etc. Owing to its wide use in food, medicine, veterinary drugs and feed additives health care products, dietary addition and cosmetics, this species has high economic and medicinal value (Wang et al., 2022a, Wang et al., 2019). Extracts from Lonicerae Flos has significant antipyretic and anti-inflammatory activities, and can exert its effects by inhibiting inflammatory factors such as NO, TNF - α, and IL-6 (Gao et al., 2021). Chlorogenic acid, isochlorogenic acid, caffeic acid, flavonoids, and triterpenoid saponins may be the active ingredient groups that exert anti-inflammatory activity in the water extract of Lonicerae Flos; These extracts have antibacterial activity and strong inhibitory activity against *Staphylococcus aureus* and *Escherichia coli.* Their active substances include phenolic acids, flavonoids, and volatile oils, among them phenolic acids have the strongest antibacterial ability (Yu et al., 2019); Moreover, Lonicerae Flos has strong antiviral effects, it inhibits herpes virus, respirvirus, H1N1 influenza, and SARS coronavirus virus. It significantly contributed to the management of COVID-19 in China (Lyu et al., 2021; Gu et al., 2021), which is potentially related to its flavonoids and organic acid components; The antioxidant effect of Lonicerae Flos is mainly related to the radical-scavenging effect of its flavonoids and chlorogenic acid contents (Wang et al., 2009). In recent years, researchers have increasingly focused on the underlying regulatory mechanisms of the active ingredients, such as chlorogenic acid, flavonoids, and triterpenoid saponins, in Lonicerae Flos. Chlorogenic acid enrichment of occurs mainly in the bud stage, and its abundance decreases with the cracking of the corolla, resulting in a significant difference in chlorogenic acid content before and after the harvest period (Pan et al., 2021). Active ingredients including flavonoids and saponins show a similar accumulation trend, revealing that secondary metabolites change dynamically during different developmental periods, which is strongly associated with corolla opening (Zeng et al., 2022). The corolla of the wild-type *LM* successively cracks within a brief harvesting period lasting 3 - 7 d, resulting in an increased harvesting costs and directly affecting the yield and quality of Lonicerae Flos. However, the Xianglei variety does not open its corolla throughout the entire development period, develops neat and rod-shaped flower buds, and includes a sufficiently long harvesting period of up to 21 days. In addition, compared with the wild-type Lonicerae Flos, the actual picking yield of the xianglei type was 80%~130% higher, and the content of the active ingredient, chlorogenic acid, was 30%~35% higher than the wild-type Lonicerae Flos (Chen et al., 2015). Further exploration is needed to clarify the accumulation pattern and the associated specific regulatory network.

Genes regulate the synthesis of compounds through the transcription and translation of metabolic enzymes. The biosynthetic pathways producing secondary metabolites involve many key enzyme-encoding genes. The transcriptome links genetic information with proteins exhibiting various biological functions, which can more comprehensively reveal the expression of global genes in specific periods or tissues of organisms. It presents a powerful approach to elucidate gene expression and is increasingly applied to the synthesis and regulatory mechanism of bioactive metabolites in medicinal plants (Zhang et al., 2018). With the rapid development of analytical techniques such as GC−MS, LC−MS, and NMR, plant metabolomics technology has significantly contributed to research on resource utilization in traditional Chinese medicine via the comprehensive analysis of the metabolites present in Chinese medicinal materials; thus the differences between wild and cultivated Chinese medicinal materials, medicinal parts, origins, harvesting periods, and different developmental stages of Chinese medicine have been explored. In addition, metabolomics analysis can be regarded as a technical means of association analysis, which can be combined with other omics analyses to characterize the gene functions involved in specific metabolic pathways of interest and can also provide supporting information for gene mining. Combined with transcriptome or proteome analysis, a network of connections between genes, metabolites, and proteins can be represented, and the regulation of downstream metabolic levels by upstream transcriptional or protein level changes can be explored. Analysis of phenotypic characteristics, which often involves correlation analysis between the abundance of metabolites and qualitative and quantitative information on phenotypic characteristics, can also be performed, which involves estimating the metabolites correlated with phenotypic changes to identify relevant substances that may regulate phenotypes. Furthermore, joint analyses based on transcriptomics and metabolomics provides an effective approach for elucidating the regulatory mechanisms associated with bioactive ingredients. Previously, this approach was used to reveal the profile and biosynthesis of caffeine in tea and coffee plants (Zhang et al., 2022), key gene networks involved in soluble sugar and organic acid metabolism during fruit development in eastern melon (Cheng et al., 2022), the regulatory network for flavor formation in *Actinidia chinensis* (Wang et al., 2022b), anthocyanin synthesis mechanisms in purple wheat (Wang et al., 2021a), and aroma formation in *Macadamia integrifolia* (Wang et al., 2022c).

The genetic breeding of medicinal plants significantly differs from traditional plant breeding. The characteristics of medicinal plants themselves are “medicinal”, hence, effective breeding requires high attention to their medicinal value and safety, as well as their phenotype and yield during the process of variety selection and cultivation. The Xianglei-type *LM* is a new variety obtained through targeted cultivation after natural variation. In this study, we used wild-type and xianglei-type *LM* to conduct targeted studies focusing on the molecular regulatory mechanisms underlying these phenotype and the metabolic pathways of active ingredients in medicinal plants through transcriptional and metabolic joint analysis. We focused on the differential accumulation of high-value active components between the two varieties of *LM* showing phenotypic differences, combining internal quality with appearance, which is in line with the requirements for the molecular breeding of medicinal plants in the future. The accumulation patterns and regulatory mechanisms of terpenoids, flavonoids, and phenolic acids in different tissues and at different stages of flower development were investigated in this study, which can potentially reveal new ideas for further exploration of the biosynthetic pathways of high-value and potent active ingredients in woody plants.

## 2 Materials and methods

### 2.1 Plant materials and pretreatment

**Figure 1:**
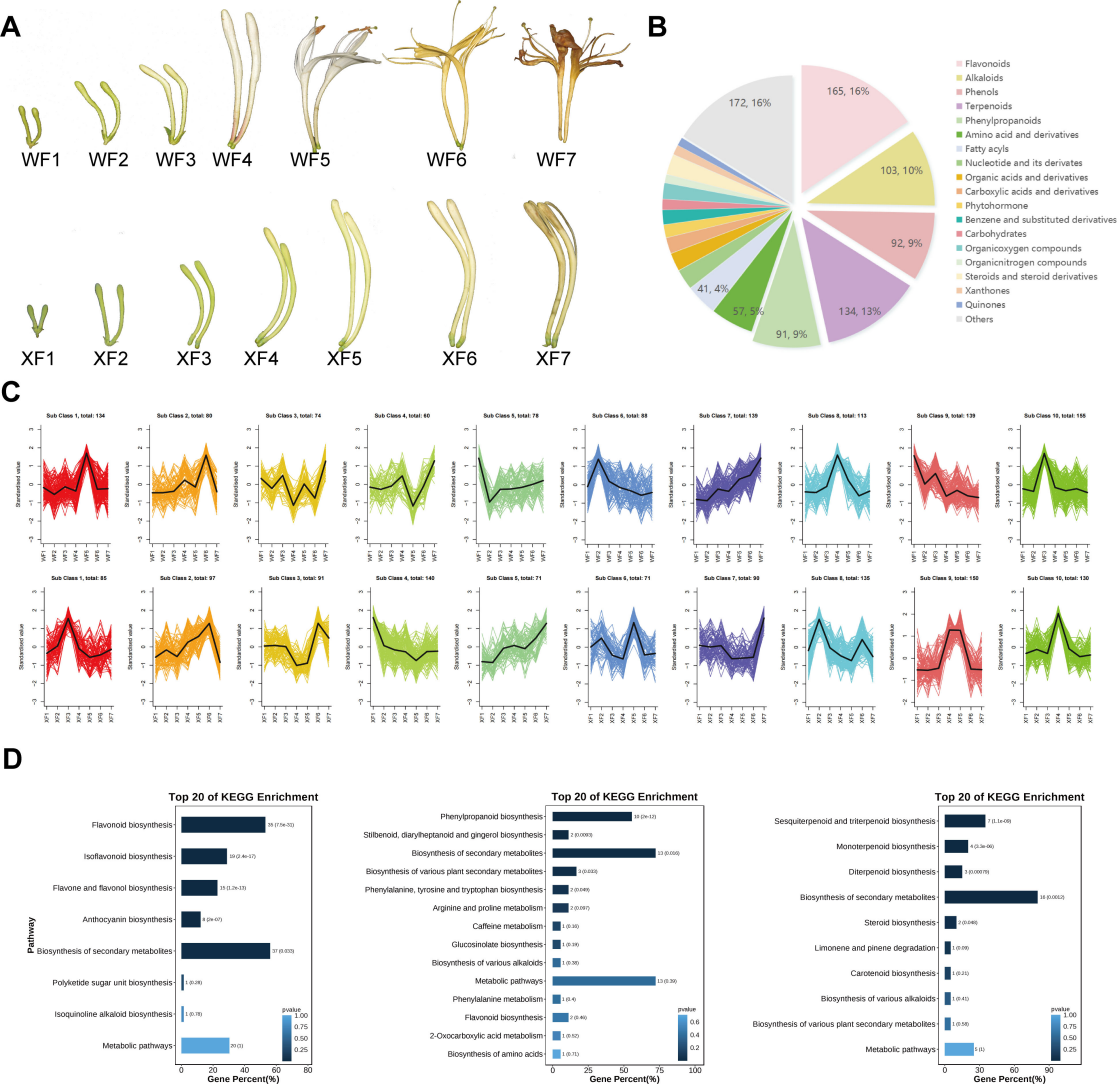
Analysis of metabolite contents in the flowers of WF and XF at different developmental stages. WF1-WF7 represent the seven developmental stages of Wild type *LM*, and XF1-XF7 represent the seven developmental stages of XiangLei type *LM*. **(A)** Morphological comparison of WT and XL *LM*. **(B)** Classes of the metabolites with annotated structures. Metabolites are divided into twenty-five categories. **(C)** Metabolite variation tendencies among the twenty cluster profiles. **(D)** KEGG pathway assignment of metabolites identified as flavonoids, phenolic acids and terpenoids of *LM*.

The plant materials, cultivated under natural conditions without exposure to extreme drought, plant diseases, and insect pests, were collected from Guanglong village (N: 27°31′, E: 110°40′; 1320 m above sea level), Shaoyang city, Hunan Province, China. For this experiment, the original flower buds of the wild-type (WT) and xianglei-type (XL) *L. macranthoides* Hand-Mazz (*LM*) at seven growth stages were selected between June and July 2022 for sampling(**Figure 1A**). Eight biological replicates of flowers from WT and XL (WF and XF) were randomly selected from the upper parts of the branches on a clear day. Three independent biological replicates were used for RNA library construction, another three biological replicates were used for UHPLC−MS/MS analysis, and the remaining samples were stored for further analysis; each biological replicate comprising flowers tissues, randomly collected from each branch, was frozen using liquid nitrogen and stored at -80 °C.

### 2.2 Extraction and detection of metabolites by UHPLC-MS/MS

Metabolite extraction and analysis were performed by Shanghai BIOTREE Biological Technology Co., Ltd. (Shanghai China). A 10 mg crushed aliquot of each freeze-dried samples were accurately weighed then transferred to Eppendorf tubes, which containing 500 μL extraction solution (75% methanol in water, precooled at -40°C, containing the internal standard 2-amino-3-(2-chloro-phenyl)-propionic acid). The resulting supernatant was filtered through a 0.22 μm microporous membrane, subsequently diluted 10-fold with the extraction solution containing internal standard, UHPLC separation was conducted via an EXIONLC system (Sciex). Chromatographic separation was achieved by injecting 2 μL sample into an Waters Acquity UPLC HSS T3 column (1.8 μm 2.1x100 mm) with the flow rate set at 400 μL/min, column temperature at 40°C and autosampler temperature at 4**°**C, respectively. The mobile phases consisted of A and B (A:0.1% formic acid in water, v/v; B:acetonitrile) Separation was conducted with the following gradient: 2% B for 0.5 min,; 2% to 50% B, in 9.5 min,; 50% to 95% B in 1min,; 95% B for 2 min,; 98% to 2% B in 0.1 min,; and 2% B over 15 min,;. The mass spectrometry analysis was performed in MRM mode using Sciex QTrap 6500+(Sciex Technologies), with temperature set at 400°C, and the curtain gas pressure set at 35 psi. The IonSpray voltage was set as 5500 for positive polarity, and 4500 V for negative polarity; The ion source gas pressure was 1:60 psi and 2:60 psi with a DP at 100** V** for both positive and negative polarity.

### 2.3 Quantitative and qualitative metabolite profiling

SCIEX Analyst Work Station Software (Version 1.6.3) was employed for all MS data collection and quantitative analysis of target compounds. The peak detection and annotation were performed via an in-house R program and MS/MS database. The relationships among samples was implemented by principal component analysis (PCA) following R package models. Afterwards, the metabolites were annotated and classified to facilitate the analysis of active ingredient biosynthesis via the KEGG compound database. Metabolites with VIP > 1, p values < 0.05 and twofold-change were regarded as significantly differentially accumulated metabolites (DAMs) between groups. The metabolite enrichment analysis and pathways analysis to which the significantly regulated metabolites mapped were integrated using MetaboAnalyst (https://www.metaboanalyst.ca/).

### 2.4 RNA sequencing and transcriptomic analysis

RNA sequencing was performed on the Illumina NovaseqTM 6000 platform, taking the total RNA extracted from frozen petals of wild-type and xianglei-type *LM*s as samples. After assembly and annotation, the transcripts per million (TPM) values were calculated via Sa*Lm*on (version 0.8.2) to quantify gene expression, and DESeq was adopted to determine the differentially expressed genes (DEGs) based on the following parameters: p value<0.05 and absolute |log2(fold-change)| ≥2. To identify the DEGs that were significantly enriched in GO terms and metabolic pathways (p value<0.05), functional analyses was accomplished by performing GO (Gene Ontology) and KEGG enrichment on the annotated DEGs.

### 2.5 Weighted gene coexpression network construction and visualization

The Weighted gene coexpression network construction based on the RNA-seq data via the WGCNA Rpackage (Zhang and Horvath, 2005; Langfelder and Horvath, 2008) was constructed to investigate the genes related to the biosynthesis of flavonoid, phenolic acid and terpenoids during flower development. The resulting adjacency matrix was converted by unsigned TOMtype using the default parameters. Eigengene values were used to calculate the correlation between metabolites and gene modules which then visualized by Cytoscape v3.9.1.

### 2.6 Cojoint analysis of the transcriptome and metabolome

The correlation coefficients among key genes and metabolites were analyzed via the Pearson’s correlation algorithm (Bishara and Hittner, 2012). The integrated genes-metabolite regulatory network was constructed based on KEGG pathway.

### 2.7 Quantitative real-time PCR evaluation

Sixteen unigenes including structural gene, hormone-related gene and TF were randomly selected for RT−qPCR evaluation with 18S as endogenous control. The gene-specific RT−qPCR primers were designed via PrimerQuest™ Tool and the sequences were listed in **Supplementary Table S12**. RT−qPCR analysis performed on the Bio-Rad CFX96 real-time PCR detection system with three biological replicates. The PCR reaction system and procedure reference to the previous papers (Long et al., 2021) and the 2^-ΔΔCT^ method (Zhao et al., 2021) was used to calculate the relative expression level of selected gene.

## 3 Results

### 3.1 Morphological characterization of the WF and XF

The wild-type and xianglei-type *LM*s were categorized into seven developmental stages (wild-type, WF1–WF7; xianglei-type, XF1–XF7) (**Figure 1A**) considering previous classification standards for flowering periods, which were based on harvest time, degree of development and use color, size, and appearance as descriptive indicators (Long et al., 2023). The corollas of the two varieties gradually grew at a different rate from the early developmental to the aging stage, the color of the flower buds significantly changed at different stages of development as well. The corolla of wild-type *LM* gradually expanded from young buds and unfolded in the fifth stage of flowering (WF5), with a color change from green to white, followed by a transformation to yellow. The corolla of XL *LM* never unfolds from young buds to withering, and the color gradually changes from green to yellow-green, finally turning yellow.

### 3.2 Metabolomic profiles of WF and XF at different flower stages

#### 3.2.1 Compound profiles of WF and XF at different flower stages

To determine the metabolite differences between WF and XF, a broadly targeted metabolomics approach were performed to constructed a metabolomics database of *LM* flower bud samples. After MRM data acquisition and processing, we identified 1060 metabolites including a total of 165 flavonoids, 103 alkaloids, 92 phenols, 134 terpenoids (including triterpenoids, diterpenoids, sesquiterpenoids, monoterpenoids and iresters), 91 phenylpropanoids (including phenylpropanoids, coumarins, and lignans), 57 amino acids and derivatives, 27 nucleotides and their derivatives, 21 carboxylic acids and derivatives, 24 organic acids and derivatives, 14 carbohydrates, 41 fatty acyls, 17 steroids and derivatives, 17 phytohormones, 19 benzene and substituted derivatives and others (**Figure 1B**, **Supplementary Table S1**). We investigated the changes in the metabolite contents during the different flower development periods of WF and XF via a circle clustering heatmap generated with the Metware Cloud(https://cloud.metware.cn, accessed on 15 Aug 2024). (**Supplementary Figure S1**, **Supplementary Table S1**).

Based on the trends in metabolite abundance changes, we identified 10 profiles in WF and XF. The profiles of the two varieties presented different compound variation clusters and trends (**Figure 1C**). Subsequently, we performed a trend analysis for all of the metabolites. Most flavonoids and phenolic acid components were enriched in profile 9 (WF) and profile 9 (XF), revealing a significant difference in trend between the two varieties. Specifically, in WFs, the flavonoids and phenolic acids contents continuously decreased with the developmental and aging processes in flowers, whereas, a high content was maintained in XF during harvest periods 4 and 5. Moreover, in both varieties, the trends of variation in terpenoids were reflected in F4 and F5, which are enriched in profiles 10 (WF) and profile 4 (XF). Profile 10 in WF showed that the accumulation of terpenoids reached its highest value in WF3, decreased after WF4, and decreased overall in profile 4 in XF, and reached its lowest level in XF5.

#### 3.2.2 High-value active ingredients in the metabolic pathway during flower development

Throughout the flower opening process in WF and XF, a consistent number of flavonoid compound species, 165 metabolites including 39 flavones, 28 flavonols, 4 flavan-3-ols, 10 anthocyanins, 16 flavanones, 9 dihydroflavonols, 21 isoflavones, 13 chalcones, 2 proanthocyanidins etc. were detected using UPLC-MS (**Supplementary Table S2**). Most anthocyanins, including delphinidin-3-O-glucosides, pelargonidin-3,5-O-diglucoside chlorides, malvidin 3-O-glucosides, malvidin 3-glucosides, delphinidin, cyanidin B2, procyanidin B1, and procyanidin B2 were highly abundant in WF4 and XF4. Additionally, 38 metabolites, including narcissoside, isoquercitrin, L-epicatechin, chalcone, genkwanin, fisetin, eriodictyol, and cianidanol, decreased during the flower development period in WFs. Conversely, 18 metabolites, including diosmetin, fisetin, taxifolin, astragalin, kaempferol-3-O-rutinoside, isosakuranetin, demethoxycapillarisin, robinetin, narirutin, and cyanidin-3-O-rutinoside chloride, etc., increased during the flower development period in XF. In summary, the accumulation of bioactive flavonoids differed between the two varieties and across different flower development processes. In this study, 43 metabolites were identified as phenolic acids and their derivatives (**Supplementary Table S2**), including cinnamic acid, shikimic acid, caffeic acid, trans-caffeic acid, chlorogenic acid, isochlorogenic acid B, ferulic acid, sinapic acid, 1,4-dicaffeoylquinic acid, and 1-caffeoylquinic acid and others. Cinnamyl cinnamate, octadecyl p-coumarate, ferulic acid, caffeic acid, and 3-hydroxycoumarin had the lowest contents in WF4 and the highest in XF4. Moreover, some phenolic acids (5-O-caffeoylshikimic acid, p-hydroxy-cinnamic acid, isochlorogenic acid B, caffeic acid, 5-hydroxyconiferaldehyde, coniferylaldehyde, 1,4-dicaffeoylquinic acid, and 3-hydroxycoumarin) accumulated mainly in WF1, WF2, and WF3, whereas in XF, they mainly accumulated in the middle and late stages (XF4, XF5, XF6, and XF7). A total of 134 terpenoids were identified (**Supplementary Table S2**), which included 55 triterpenoids, 26 diterpenoids, 21 sesquiterpenoids, 21 monoterpenoids, 9 iridoids and other terpenes. Among them, triterpenoids and their glycosides are characteristic components of *LM*, which were primarily abundant in flower buds, with macranthoidin B and dipacoside B serving as important factors for screening the quality markers of *LM*. Triterpenoids, including esculentoside B, taraxerol, lupenone, glycyrrhetinic acid, ganoderic acid Jb, ganoderic acid H, betulin, and asiatic acid, accumulate mainly in prostages F1, F2 and F3. Iridoids and their glycoside compounds (valazine, geniposide, genipin, swertiamarin, agnuside, oleuropein, oleuroside, pecoside, and sweroside) are the main water-soluble components in *LM* plants, which primarily accumulated in the middle and late stages (F4~F7) in XF and the early and middle stages (F1~F5) in WF.

Furthermore, Kyoto Encyclopedia of Genes and Genomes (KEGG) analysis was performed to identify key metabolites (p value <0.05) involved in metabolism of high-value active ingredients in *LM* (**Figure 1D**). In this study, 165 flavonoids involved in the flavonoid biosynthetic pathway were significantly enriched in Flavonoid biosynthesis, ko00941(35), Isoflavonoid biosynthesis, ko00943(19), Flavone and flavonol biosynthesis, ko00944(15), Anthocyanin biosynthesis, ko00942(8), Biosynthesis of secondary metabolites, ko01110(37); 43 phenolic acids and derivatives involved in the phenolic acid biosynthetic pathway were significantly enriched in Phenylpropanoid biosynthesis, ko00940 (10), Stilbenoid, diarylheptanoid and gingerol biosynthesis, ko00945(2), Biosynthesis of secondary metabolites, ko01110(13), Biosynthesis of various plant secondary metabolites, ko00999(3), Phenylalanine, tyrosine and tryptophan biosynthesis, ko00400(2); 134 terpenoids involved in the terpenoid biosynthetic pathway (including Triterpenoids, Diterpenoids, Sesquiterpenoids and Monoterpenoids and Iridoids) were significantly enriched in Sesquiterpenoid and triterpenoid biosynthesis, ko00909(7), Monoterpenoid biosynthesis, ko00902(4), Diterpenoid biosynthesis, ko00904(3), Biosynthesis of secondary metabolites, ko01110(16), Steroid biosynthesis, ko00100(2).

### 3.3 Functions of the DAMs involved in WF and XF flower development

**Figure 2:**
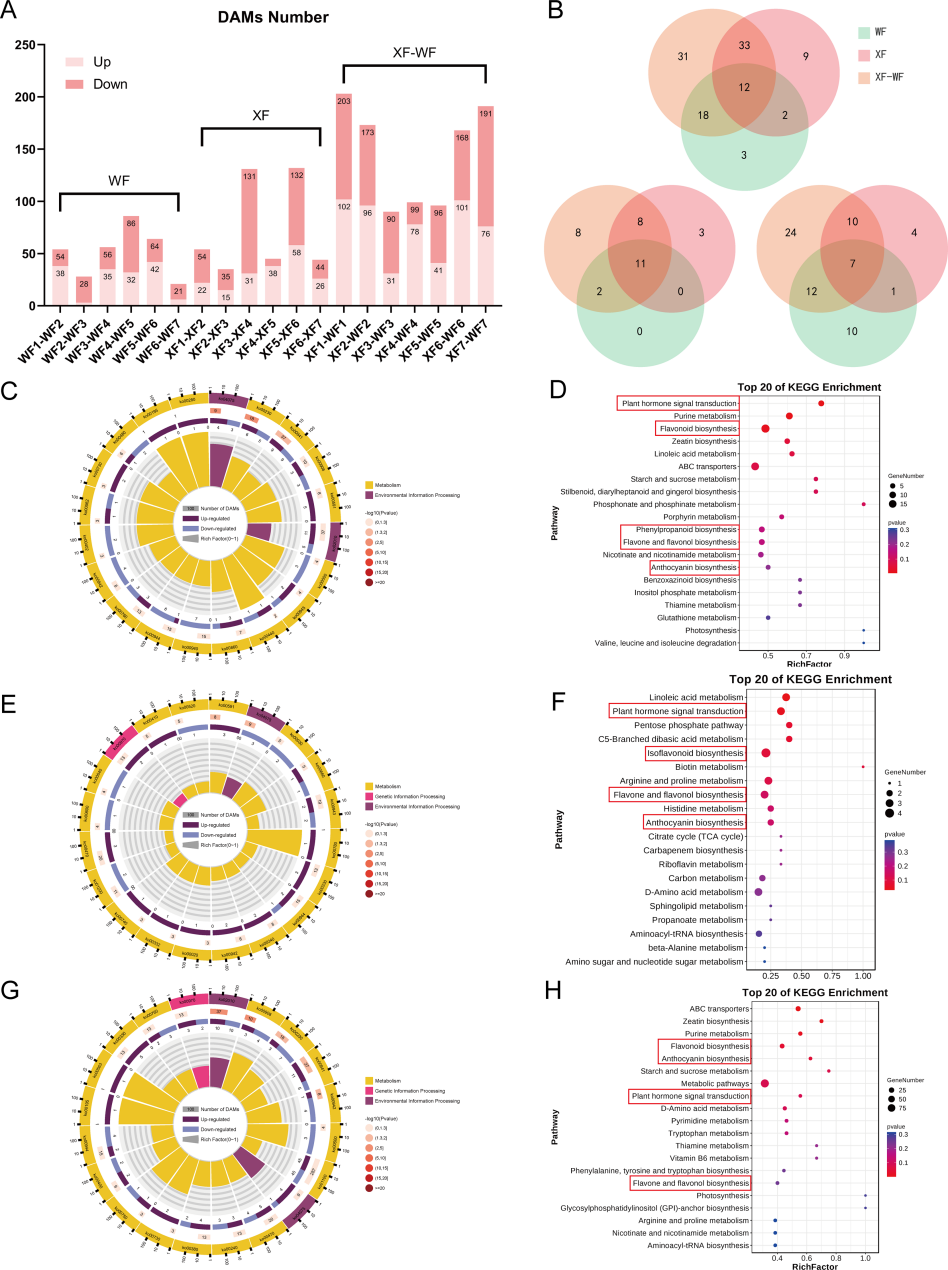
Functional analysis of the DAMs involved in WF and XF flower development. **(A)** DAMs number in all compare groups. **(B)** Venn analysis of differently accumulate flavonoids, phenolic acids and terpenoids in WF, XF, and XF-WF compare groups. **(C)** KEGG Ontology enrichment circle diagram and **(D)** KEGG enrichment analysis of DAMs for pre-floral development stage XF123 vs. WF123. **(E)** KEGG Ontology enrichment circle diagram and **(F)** KEGG enrichment analysis of DAMs for mid-floral development stage XF45 vs. WF45. **(G)** KEGG Ontology enrichment circle diagram and **(H)** KEGG enrichment analysis of DAMs for late-floral development stage XF67 vs. WF67.

Next, VIP>1, p-value < 0.05 and two-fold change were used to determine differentially accumulated metabolites (DAMs) between the groups (**Figure 2A**). A total of 647 significant DAMs were subsequently grouped into eight clusters via Mfuzz clustering based on their content (**Supplementary Figure S6**). When the number of DAMs was compared with that of both WF and XF for each flower development stage, venn diagrams were generated, which revealed that 12 flavonoids, 11 phenolic acids and 7 terpenoid compounds accumulated differently between the wild and xianglei variety and different flowering periods (**Figure 2B**). The number of DAMs in the F3, F4, and F5 comparison groups was only half of that in F1, F2, F6, and F7, indicating that the differences in metabolic accumulation between the two varieties of *LM* were mainly in the early and late stages of flower development, which is consistent with the PCA results. The samples were further divided into the prefloral (F1, F2, and F3), mid-floral (F4 and F5), and late-floral (F6 and F7) development stage for differential analysis and KEGG analyses. KEGG pathway enrichment analyses. revealed that 566 DAMs were annotated. The flavonoid biosynthesis, arginine and proline metabolism, ABC transporter, plant hormone signal transduction, nucleotide metabolism, purine metabolism, and D-amino acid metabolism pathways were significantly enriched (**Figures 2C–H**).

### 3.4 Transcriptome analysis of WF and XF at different flower stages

After removing low-quality reads, ambiguous reads, and adapter sequences, we initially generated 259.93 Gb of data from 42 libraries, and each library yielded between 33.05 and 57.26 million clean reads (**Supplementary Table S3**). The Q20%, Q30%, and GC% results indicated that the throughput and sequencing quality were sufficiently high to warrant further analysis (**Supplementary Table S4**). After ***de novo*** assembly, a total of 107,737 unigenes with an N50 of 1602 bp were detected in the flower samples (**Supplementary Table S4**). In addition, the assembled 107,737 sequences were all annotated in the database, and 51757, 33397, 45457, 45742, 56402, and 47389 unigenes were annotated respectively (**Supplementary Table S5**).

**Figure 3:**
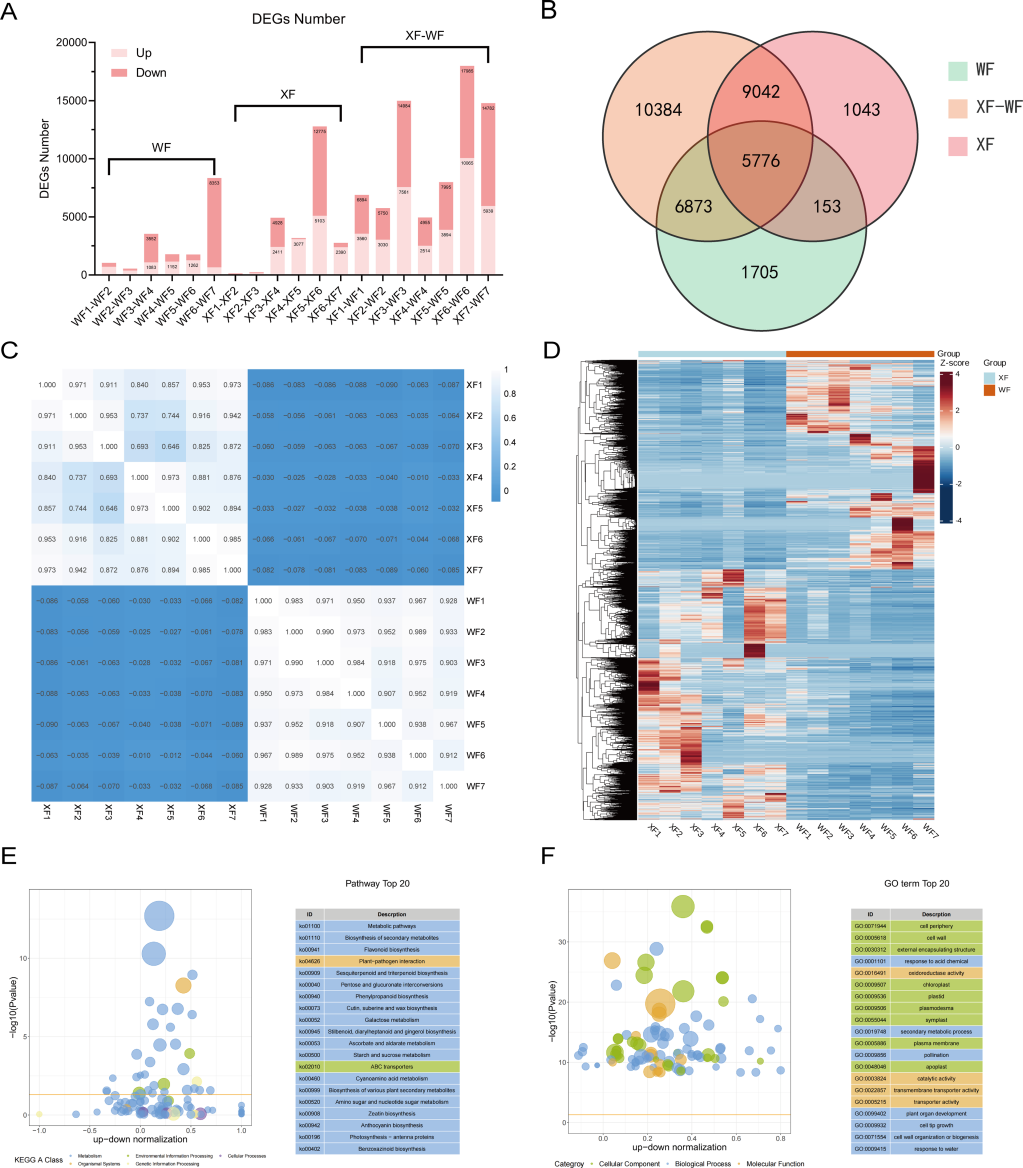
Transcriptome sequencing of WF and XF samples at seven stages. **(A)** Number of DEGs in all compared groups. **(B)** Venn diagram of DEGs associated with WF, XF, and XF-WF. **(C)** Correlation heatmap of 5,776 common DEGs. **(D)** Heatmap of the transcriptome expression of 5,776 common DEGs. **(E)** KEGG pathway assignment of all DEGs among WF and XF. **(F)** GO classification of all DEGs among WF and XF.

Through differential expression analysis between the two *L. macranthoidesis* varieties at different flowering periods, a total of 34976 DEGs were identified between the sample groups, with the number of DEGs ranged from 137 to 17985 (**Figure 3A**). There were 14,507 DEGs in the seven WF comparison groups, 16,014 DEGs in XF, and 32,075 DEGs in XF vs. WF. Compared to WF, there were more DEGs in XF, and most of them showed an upward trend, which was reflected in each comparison group. Furthermore, among the WF, XF, and XF vs WF groups, 5,776 common DEGs were indicated using Venn diagrams (**Figure 3B**). These common 5,776 DEGs exhibited varied expression patterns, showing a closer relationship(**Figures 3C, D**). Among the WFs, the most abundant DEGs were found in the WF6 vs. WF7 comparison group, whereas those in the XF5 vs XF6 comparison group were in the XF. However, the number of DEGs in the early bud (F1 and F2) comparison between the two varieties was significantly lower, suggesting that the two varieties differed significantly in terms of middle and late flower development.

KEGG-based enrichment analysis of all the DEGs revealed the top 20 KEGG pathways which were significantly enriched (**Figure 3E**). These pathways included metabolic pathways (ko01100), biosynthesis of secondary metabolites (ko01110), flavonoid biosynthesis (ko00941), terpenoid backbone biosynthesis (ko00900), sesquiterpenoid and triterpenoid biosynthesis (ko00909), phenylpropanoid biosynthesis (ko00940), etc. GO enrichment analysis of these DEGs revealed that they were widely distributed in three functional groups, i.e., biological processes, molecular functions, and cellular components (**Figure 3F**). According to the GO analysis, the response to acid chemical was the largest group of biological processes. The genes related to “cell periphery”, “cell wall”, and “external encapsulating structure” were predominant in the category of “cellular components”. In the molecular function category, the genes were mostly involved in oxidoreductase activity, catalytic activity, transmembrane transporter activity, and transporter activity.

### 3.5 Integration of related genes and metabolites involved in flavonoid, phenolic acid and terpenoid biosynthesis during WF and XF flower development

**Figure 4:**
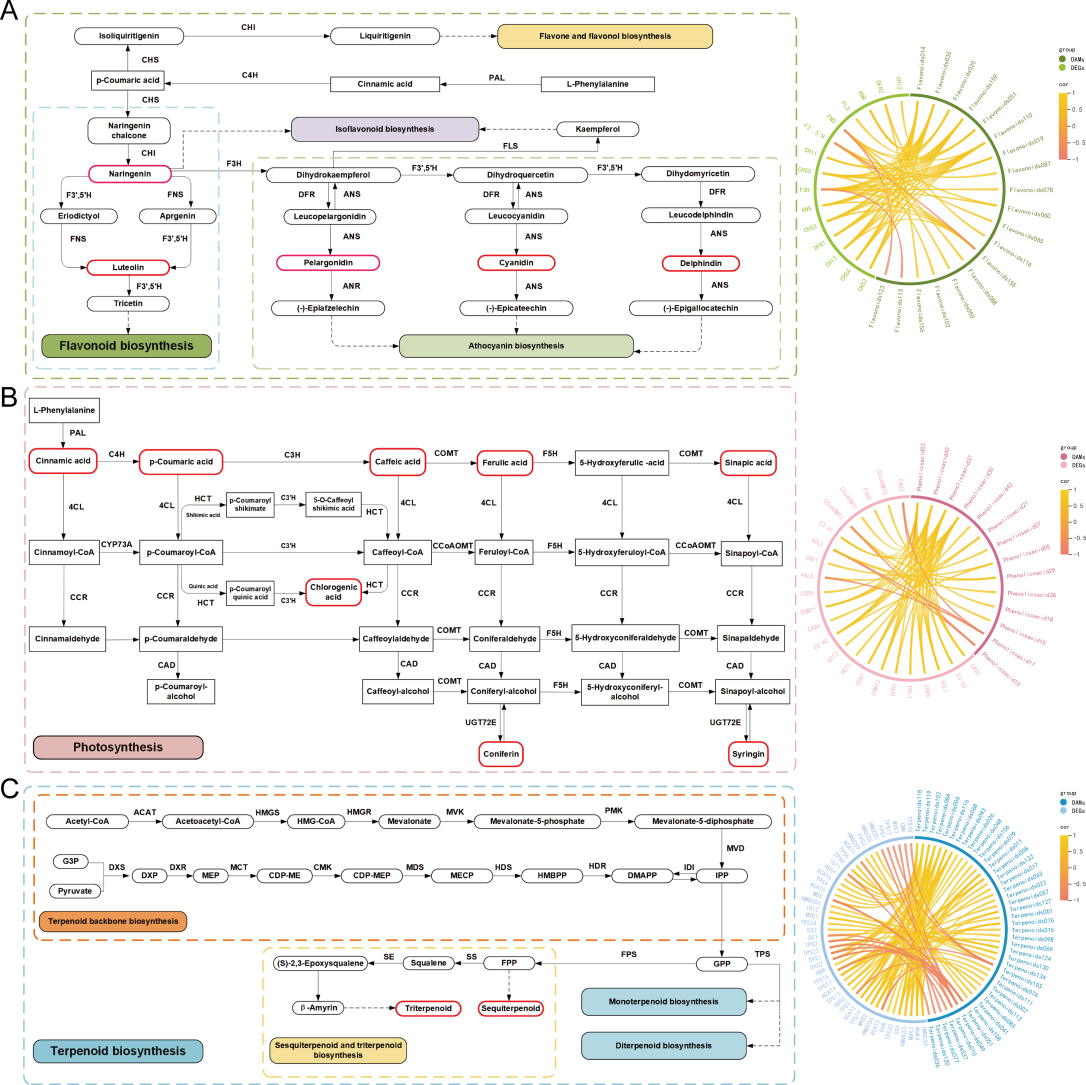
Integration of related genes and metabolites involved in flavonoid, phenolic acid and terpenoid biosynthesis The left column represents the proposed pathways for flavonoid **(A)**, phenolic acid **(B)**, and terpenoid **(C)** biosynthesis in *LM*. The right column represents the correlation network between structural genes and metabolites.

To reveal the molecular mechanisms underlying the different accumulations of active ingredients in *LM* at different stages, we conducted transcriptome sequencing and correlated the results with the metabolomic data. We identified 128 enriched structural genes that regulate 34 enzymes (CHS, CHI, FNS, F3’,5’H, FLS, F3H, DFR, ANS, ANR, PAL, C4H, 4CL, CCR, CAD, HCT, C3’H, F5H, COMT, CCoAOMT, ACAT, HMGCS, HMGCR, MVK, MVD, IDI, DXS, DXR, MCT, CMK, MDS, HDS, HDR, TPS, FPS, SS, and SE), of which 16 genes regulate 9 enzymes involved in flavonoid biosynthesis, anthocyanin biosynthesis, flavonoid and flavonol biosynthesis, and isoflavonoid biosynthesis; 46 genes regulate 8 enzymes involved in phenolic acid biosynthesis; and 66 genes regulate 17 enzymes involved in the biosynthesis of terpenoid backbone biosynthesis and sesquiterpenoid and triterpenoid biosynthesis (**Supplementary Table S6**). To identify the genes closely related to flavonoid, phenolic acid and terpenoid biosynthesis, all 128 enriched structural genes (DEGs) and differentially accumulated metabolites (DAMs), including 165 flavonoids, 34 phenolic acids, and 134 terpenoids detected at different flower developmental stages, were selected for integrative analysis. Using Pearson’s correlation analysis, we evaluated the correlation coefficients (r ≥ 0.8) between the DEGs and DAMs during flower development (**Figure 4**). In flavonoid biosynthesis, 2 *CHI*s (TRINITY_DN46726_c4_g1, TRINITY_DN43583_c0_g1), *ANS*s (TRINITY_DN28844_c0_g1), 4 *CHS*s (TRINITY_DN46824_c0_g4, TRINITY_DN46824_c0_g2, TRINITY_DN46824_c0_g5, TRINITY_DN46824_c0_g6), *DFR*s (TRINITY_DN48211_c1_g5), and *F3H*s (TRINITY_DN41729_c3_g1) were positively correlated with hesperetin, chalconaringenin, naringenin, amentoflavone, kaempferol, safflor yellow A, and butin. Nine candidate DEGs, including *PAL* (TRINITY_DN41142_c2_g1), *4CL* (TRINITY_DN44156_c3_g1), *C3’H* (TRINITY_DN50056_c3_g3), 3 *CCR* (TRINITY_DN48406_c0_g4, TRINITY_DN53148_c0_g2, TRINITY_DN53072_c0_g4), *CAD* (TRINITY_DN42601_c1_g4), *COMT* (TRINITY_DN54977_c4_g2), and *HCT* (TRINITY_DN44906_c4_g2), were identified, which are positively connected by 5 phenolic acid compounds (isoferulic acid, methyl caffeate acid, dihydroconiferyl alcohol, and syringarin). Furthermore, 3 *ACAT*, *DXR*, *HDS*, 3 *HMGCS*, *MDS*, *MVD*, 2 *MVK*, *SS*, and 5 *TPS* genes were identified as key genes correlated with 14 terpenoids involved in terpenoid biosynthesis. Among them, 30 pairs were positively correlated, and 2 pairs were negatively correlated. To further explore the mechanism of flavonoid, phenolic acid, and terpenoid accumulation in *LM*s, markedly upregulated or downregulated DAMs and key structural genes were mapped onto the flavonoid, phenolic acid, and terpenoid metabolic pathways, which are depicted in **Figures 4A–C**, respectively. The expression levels of the genes encoding enzymes that catalyze each step of the biosynthetic pathway are shown in **Supplementary Figure S4**.

### 3.6 Hormone-related genes involved in the biosynthesis of active ingredients

**Figure 5:**
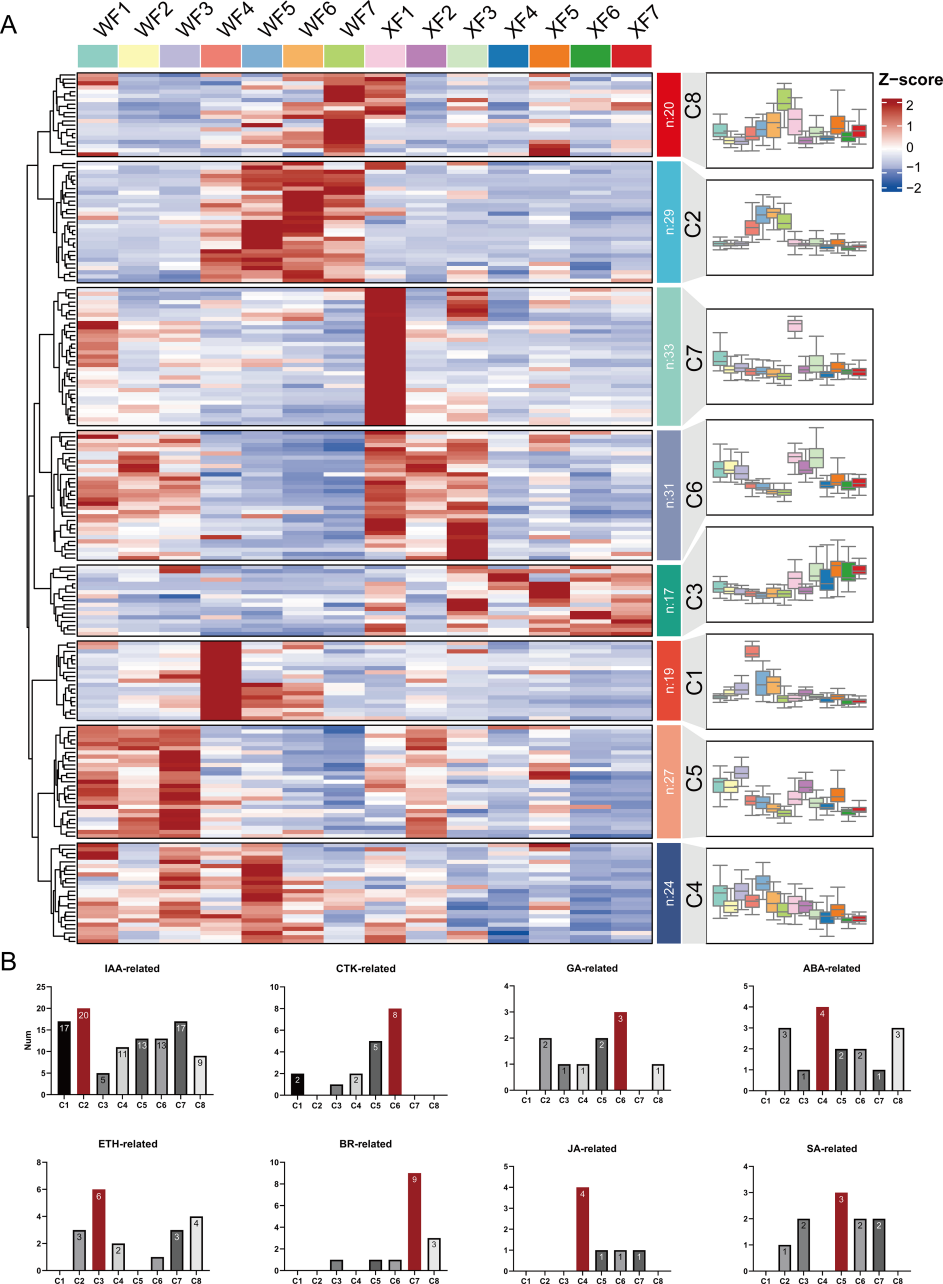
Hormone-related genes involved in the biosynthesis of active ingredients. **(A)** Cluster analysis of 200 hormone-related genes. **(B)** Bar graph of the distribution of genes in the eight clusters.

Hormone-related genes, including 105 IAA-related genes (auxin), 18 CTK-related genes (cytokinin), 10 GA-related genes (gibberellin), 16 ABA-related genes (abscisic acid), 19 ETH-related genes (ethylene), 15 BR-related genes (brassinosteroid), 7 JA-related genes (jasmonic acid), and 10 SA-related genes (salicylic acid), were further analyzed to explore the role of plant hormones in the biosynthesis of active ingredients at different developmental stages of *L. macranthoides*,. The results revealed 77 genes exhibiting significantly differentially expression between the two breeds, with IAA-related genes accounting for 59.7%. The heatmap (**Supplementary Figure S5**), cluster profiles (**Figure 5A**) and bar chart (**Figure 5B**) based on relative expression show the distributions of these hormone-related genes in eight different expression trend gene sets. Subsequent trend analysis revealed that a total of the 200 genes were distributed across 8 clusters. Clusters 1 (19 genes) and 7 (33 genes) were highly expressed only in WF4 and XF1, respectively; Clusters 2 (20 genes) and 8 (20 genes) were highly expressed mainly in WF4, 5, 6, 7, and the middle and late flower stages in WF. Conversely, 17 genes in Cluster 3 were highly expressed mainly in XF4, 5, 6, 7, and the middle and late flower stages in XF; the expression of 24 genes in Cluster 4 was significantly lower in XF than in WF; notably, the expression of 31 genes in Cluster 6 showed the same trend in both WF and XF, which was highly expressed in early flowering stages F1, 2, and 3 but decreased in the middle and late stages; 27 genes in Cluster 5 presented similar trends in WF. The enrichment results indicate that IAA-related genes (*ARF/AUX/GH3/IAA/SAUR/LAX*) were enriched mostly in Clusters 1, 2 and 7, which play a role mainly in the late stage of flower development, with some playing a specific role in XF1; CTK-related genes (*AHP/AHK/ARR*) were enriched mostly in Clusters 5 and 6, indicating potential functions in the early stage of flower development; 11 ETH-related genes (*CTR1/EIN2/ERS1/ETR/ERF/EIL/MKK*) were enriched in Clusters 3 and 8, showing high expression in the middle and late stages (F4, 5, 6, 7) of WF and XF, closely related to their function in flower maturation and aging processes; and GA-related genes (*GID/GAI*), ABA-related genes (*ABF/PP2C/PYL*), BR-related genes (*BAK/CYCD/BRL*), JA-related genes (*MYC2/JAZ/COI1*), and SA-related genes (*NPR/TGA/TGAL*) presented expression trends in Clusters 6, 4, 7, 4, and 5, respectively. Cluster analysis revealed a close connection between these genes and *LM* flower development. However, these findings reflect the direct or indirect regulation of hormone-related genes and the accumulation of active substances during flower development in *LM*.

### 3.7 Identification of gene modules associated with key metabolite pathways using WGCNA correlation networks

**Figure 6:**
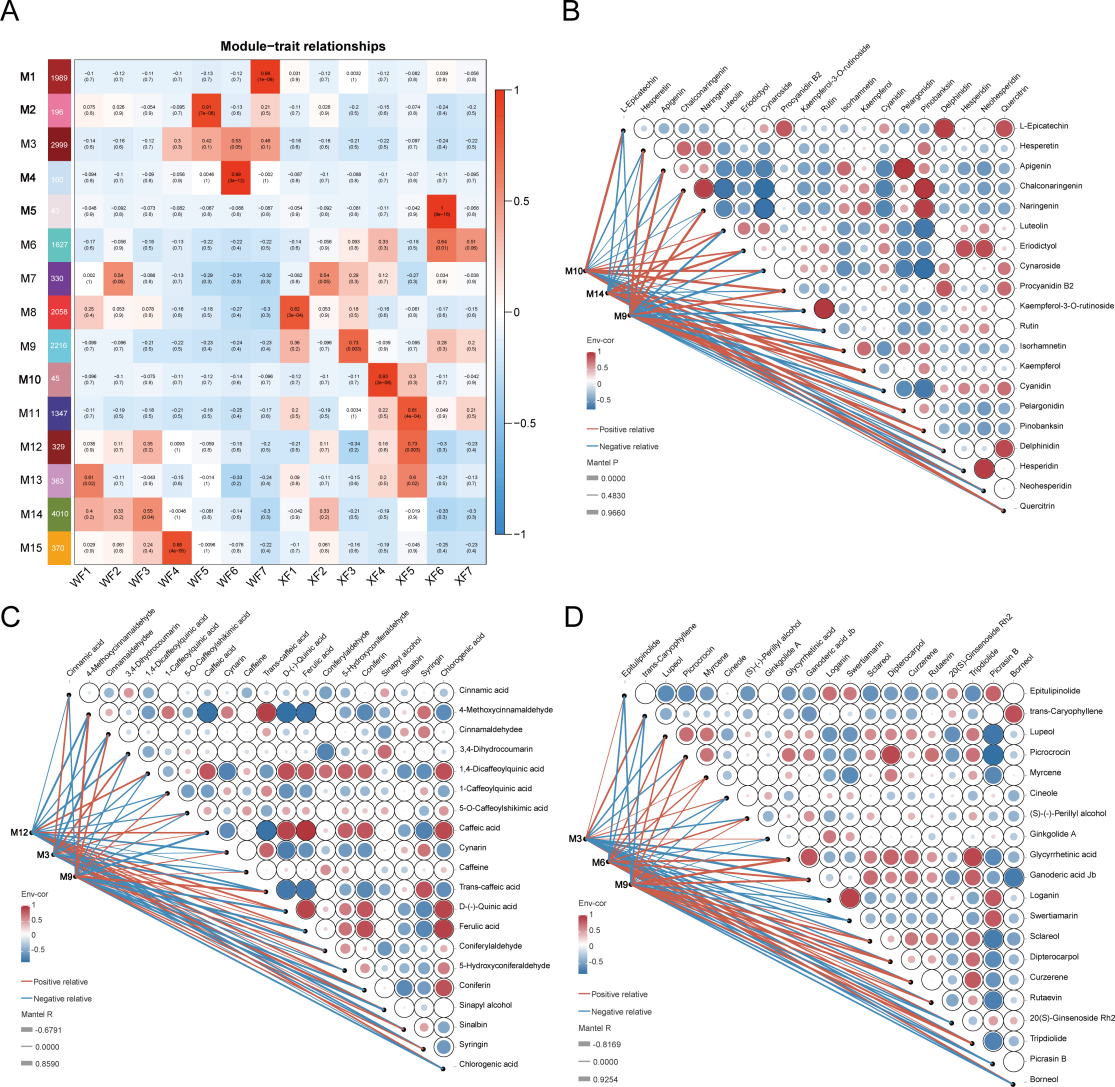
Weighted gene coexpression network analysis (WGCNA) of the relative DEGs. **(A)** Construction of the coexpression modules of genes. 15 modules labeled different colors represent gene set with different expression patterns. The correlation coefficient between the module and the flower stage displayed in the cell at the row−column intersection according to the color scale on the right. **(B)** Correlation analysis between 20 key flavoids and gene modules. **(C)** Correlation analysis between 20 phenolic acids and gene modules. **(D)** Correlation analysis between 20 terpenolids and gene modules.

For coexpression network analysis, the WGCNA package was used. After filtering, the top 50% of genes with minimal MADs, outlier genes and samples were removed. We obtained a total of 18,082 genes to construct the module. Modules with a distance of less than 0.5 were merged, and a total of 15 modules were generated (**Figure 6**). The gray module represent a gene set that cannot be attributed to any module. Five coexpression modules, including M1, M2, M4, M5, and M10, were specifically correlated with individual flower stages. Among them, M4 and M5 were strongly correlated with WF6 (r = 0.99) and XF6 (r = 1), respectively, indicating that the gene sets in these modules are specifically expressed in their corresponding stages. In addition, analysis revealed that more than 80.5% of the key structural genes were duplicated in the WGCNA modules. Subsequently, Pearson correlation analysis revealed that modules M9, M10, and M14 were co-expressed with 20 abundant metabolites related to flavonoid biosynthesis; modules M3, M9, and M12 were coexpressed with 20 abundant metabolites related to phenolic acid biosynthesis. Modules M3, M6, and M9 were co-expressed with 20 abundant metabolites related to terpenoid biosynthesis. These findings indicated that genes related to the regulation of key metabolite accumulation in these modules had the strongest correlation. Furthermore, we mapped a regulatory network of TFs and key structural genes, revealing that the expression of key structural genes (such as *CHS*, *CHI*, *F3H*, *DFR*, *ANS*, *PAL*, *4CL*, *COMT*, *ACAT*, *HMGCR*, *MVK*, *DXR*, and *TPS*) was closely associated with TF families (such as *MYB*s, *ARF*s, *bHLH*, *GRAS*s, *HD-ZIP*s, and *B3*) (**Supplementary Figure S6**).

### 3.8 Validation of representative flower development-related hub gene expression

**Figure 7:**
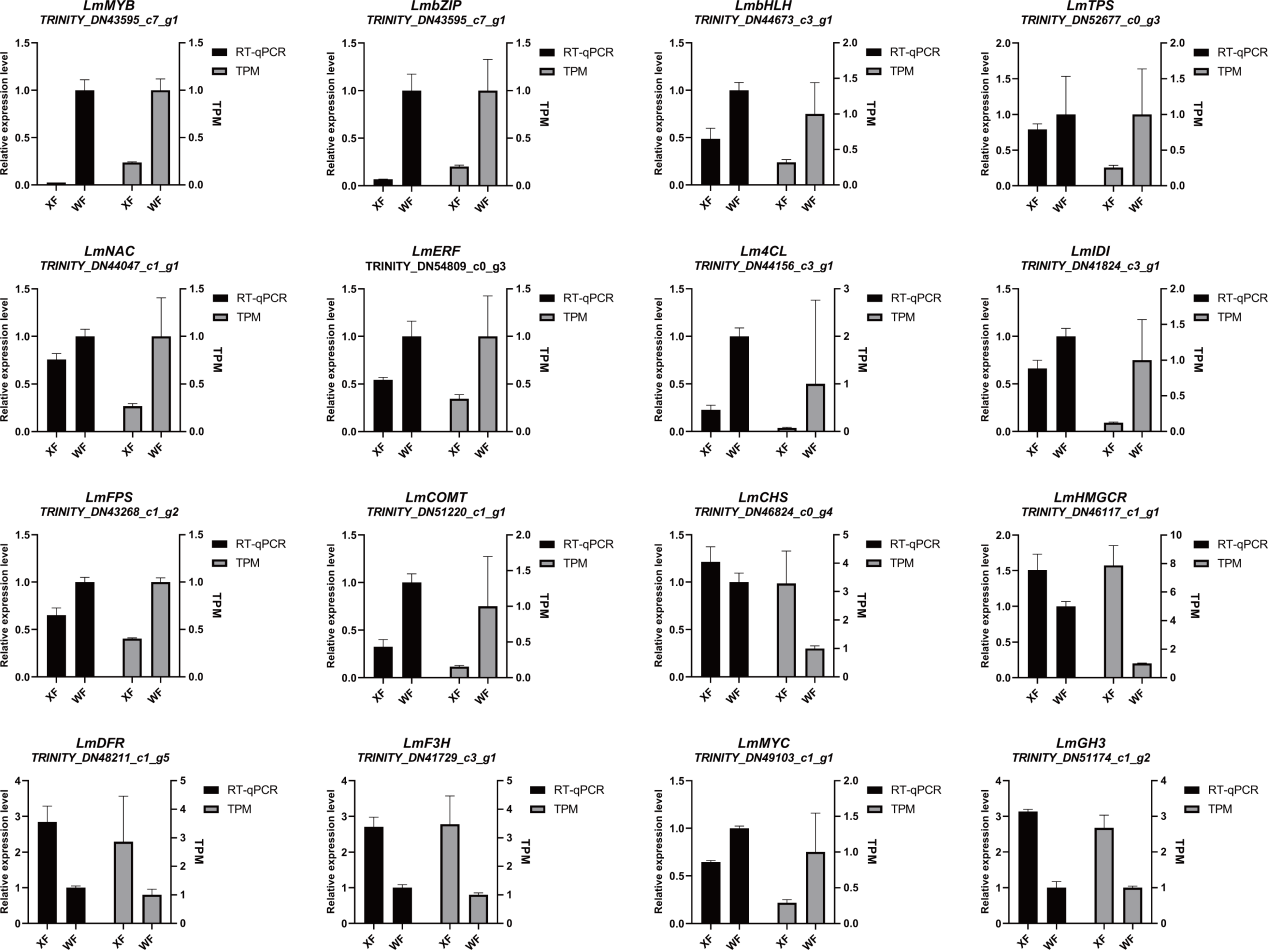
Quantitative real-time(RT−qPCR) and RNA−seq(TPM) analysis of genes involved in metabolite pathways. Relative expression levels of transcription factors and structural genes are shown as the means ± standard deviations of three biological replicates.

To confirm the RNA-seq results, RT−qPCR was performed to evaluate the expression of 16 genes involved in the biosynthetic pathway. Our observations revealed that *MYB* (TRINITY_DN43595_c7_g1), *bZIP* (TRINITY_DN56880_c3_g1), and *bHLH* (TRINITY_DN44673_c3_g1) TFs were significantly upregulated in WFs, and exhibited the lowest expression levels in XF (**Figure 7**). The RT-qPCR results of the structural genes significantly correlated with the RNA-seq results. The expression levels of *CHS* (TRINITY_DN46824_c0_g4), *HMGCR* (TRINITY_DN46117_c1_g1), *DFR* (TRINITY_DN48211_c1_g5) and *F3H* (TRINITY_DN41729_c3_g1) in XF were 2 to 3 fold higher than those in WF, indicating that these genes are potentially involved in the regulation of biosynthetic pathways. Additionally, we detected the expression levels of the JA-related gene *MYC* (TRINITY_DN49103_c1_g1) and the IAA-related gene *GH3* (TRINITY_DN51174_c1_g2) in WF and XF, which was accurate. These findings indicated that the relative expression levels were generally in line with the RNA-Seq data, confirming the accuracy and reliability of the transcriptomic profiling results.

## 4 Discussion

### 4.1 Variations in flavonoid contents during the developmental cycle of *LM*

Flavonoids are widely present in most green plants and play essential roles in plant growth, development, and stress response processes. For a long time, flavonoids have long been considered indispensable sources of nutrients and medicinal components for the human body (Roy et al., 2022; Yao et al., 2004). In this study, we analyzed 165 flavonoids among the seven stages of flower development in wild-type and Xianglei-type *LM*s, demonstrated that more than half of the compounds showed greater accumulation in XF than in WF. Most anthocyanins, including delphinidin-3-O-glucoside, pelargonidin-3,5-O-diglucoside chloride, malvidin 3-O-glucoside, malvidin 3-glucoside, delphinidin, cyanidin, procyanidin B1, and procyanidin B2, were highly abundant in WF4 and XF4. The variation and high accumulation of anthocyanins in the F4 stage may crucially regulate the development of the yellow color in *LM*s, resulting in phenotype changes in color during the development of floral organs. Plant flavonoids biosynthesis begins with the phenylpropane biosynthesis pathway, which is regulated by multiple key enzymes (Liu et al., 2021). Naringenin is a key precursor substance for the biosynthesis of many flavonoid components. Under the catalysis of CHS and CHI, p-coumaroyl-CoA produces flavonoids, including naringenin, quercetin, and chalconingenin, whose levels decrease consistently. In the pathway, the ionic abundances of the terminal compounds in the pathway, including rutin, luteolin, cynaroside, and tricetin initially increased and then peaked at F4 and F5 before decreasing, mirroring the trend of their upstream counterparts. This may be induced by the reduced accumulation of secondary metabolites upstream of the pathway, which redirects metabolic ion flow and consequently increases the accumulation of downstream metabolites. Correlation analysis revealed that key rate-limiting enzyme-encoding genes, such as *PAL*, *CHS*, *CHI*, *FNS*, *ANS* and *ANR*, are involved in the metabolism of flavones and CQA in *LM*. We detected significant differences in the expression of structural genes, which are similar to those involved in the metabolic accumulation of flavonoids. Among them, F3’,5’H (TRINITY_DN50067_c0_g1) was significantly elevated in the WF4, WF5, and WF6 stages but was stable at relatively low levels in XF, which reflected consistency with the accumulation patterns of rutin, luteolin, cynaroside, and tricetin. *ANS* (TRINITY_DN28844_c0_g1) and *ANR* (TRINITY_DN37089_c0_g1) was more highly expressed in the prophase stages of F1, F2, and F3, which was accompanied by an increased abundance of anthocyanins at F4 and F5. These findings indicate that these genes play important roles in regulating the accumulation of secondary metabolites during different developmental periods. However, their mechanisms of action and regulatory mechanisms require further investigation. Additionally, our network analysis revealed that the TFs that presumably regulated flavonoids in *LM*s, among which 25 *bHLH*s and 24 *MYB*s were enriched. Numerous studies have shown that the *MYB* and *bHLH* transcription factors can act as transcriptional repressors or activators that regulate plant growth, development, and stress response processes by mediating of flavonoid biosynthesis. For instance, the overexpression of *CmMYB8* in *Chrysanthemum morifolium* significantly inhibits the expression of genes such as *CHS, CHI, F3H, F3’H*, and *DFR*, and significantly downregulates components such as rutin, isorhamnetin, quercetin, and kaempferol (Wang et al., 2021b; Zhu et al., 2020). After the transformation of *Erigeron breviscapus EbbHLH*80 into tobacco (*N. tabacum*), the total flavonoids content in tobacco was significantly increased, resulting in the differential enrichment of 98 flavonoid components, and significant upregulation of gene expression including *CHS, CHI, F3H, FLS, ANS, and DFR* (Gao et al., 2023). MYBs typically form complexes with bHLH and WD40 proteins to regulate gene transcription. The expression of key structural genes in rice (*O. sativa*) is effectively activated by MBW members, impacting the anthocyanin content (Sun et al., 2022). These results indicate variable TF associated complex regulatory mechanisms underlying flavonoid accumulation, and the specific mechanism. Further research can validate the synergistic regulations of different active components in *LM*.

### 4.2 Variation in phenolic acids during the developmental cycle of *LM*

Phenolic acids are the main active ingredient of *LM* with multiple therapeutic and pharmacological activities such as cardioprotective, antioxidant, antiviral, antibacterial, anti-inflammatory, and antitumor activities, which are closely associated with the traditional effects of clearing away heat and detoxification (Chen et al., 2024). Our previous findings suggest that the content of chlorogenic acid accounts for over 62% of the total phenolic acid content in *LM* flowers and that the contents of chlorogenic acid, isochlorogenic acid A, and isochlorogenic acid C comprise over 90% of the total phenolic acid content (Hu et al., 2016). In the Chinese Pharmacopoeia 2020, it is considered as an important quality control indicator for the medicinal herb Lonicerae Flos (Chinese Pharmacopoeia Commission, C.P, 2020). The phenolic acid components in *LM* are mostly isomers of chlorogenic acid; the primary metabolites of chlorogenic acid include caffeic acid, quininic acid and ferulic acids; the products of further metabolism are mostly small molecules, such as catechin and hydroxybenzoic acid derivatives. We identified 43 phenolic acid compounds in the two varieties of *LM*. During flower development, the levels of most phenolic acid compounds, including 5-O-caffeoylshikimic acid, caffeic acid, quinic acid, and ferulic acid, as well as methylcinnamate, peaked in F4. The content of isochlorogenic acid B was the highest in F1 and then decreased. Chlorogenic acid gradually increased from F1, reaching its highest level at F4, and then gradually decreased. The isochlorogenic acid A content changed irregularly and then gradually decreased. The variation trend of phenolic acid content during the development of *LM* flowers varied depending on the types of phenolic acid components, which may be related to their mutual transformation. In plants, phenolic acids are primarily obtained via the phenylpropanoid biosynthetic pathway. PAL, C4H, and 4CL enzymes contribute to the synthesis of various phenolic acids (Białecka-Florjańczyk et al., 2018). Different phenolic acid derivatives are synthesized by the CAD, CCR, and COMT enzymes, and HCT acts as a key rate-limiting enzyme in CGA metabolism, suggesting that phenolic acid biosynthesis genes are closely associated with phenolic acid accumulation. In this study, all 46 key genes (*PAL, C4H, 4CL, HCT, CAD, CCR, COMT, F5H*, and *C3’H*) and TFs (r > 0.80) were found to be differentially expressed at seven developmental stages in WT and XL. These TFs, including 9 *NAC*s, 6 *MYB*s, 6 *bHLH*s, 3 *ERF*s, 6 *WRKY*s, 10 *C2H2*s and other family members, have been reported to regulate the biosynthesis of phenolic acids such as chlorogenic acid and salvianolic acid in response to hormone signaling (Liu et al., 2022; Cao et al., 2021; Shi et al., 2022; Guan et al., 2023). According to the correlation network analysis, the key rate-limiting enzymes *LmPAL*1 (TRINITY_DN41142_c2_g1) and *LmPAL*2 (TRINITY_DN52595_c1_g2) were positively regulated by *LmMYB*37 (TRINITY_DN55478_c3_g1) and *LmWRKY*12 (TRINITY_DN47707_c4_g4); *LmbHLH*57 (TRINITY_DN56449_c0_g1), *LmNAC*12 (TRINITY_DN48409_c3_g8), *LmNAC*16 (TRINITY_DN49974_c1_g3) and *LmWRKY*13 (TRINITY_DN48226_c2_g2) negatively regulated the *Lm4CL*1 (TRINITY_DN44156_c3_g1), *LmCCR*6 (TRINITY_DN53148_c0_g2) and *LmCOMT*2 (TRINITY_DN54977_c4_g2) in *LM*. These TFs are hypothesized to regulate the expression of structural genes through activation or inhibition; hence, they potentially participate in the biosynthesis and accumulation of phenolic acids in *LM*.

### 4.3 Variations in terpenoids during the developmental cycle of *LM*

Previous research indicates that triterpenes and their glycosides are characteristic compounds of *LM*. More than 50 triterpenoid saponins and their aglycones have been identified in plants belonging to the *Lonicera* genus (Fang et al., 2020). Structural analysis identified dipsacoside B, macranthoidin B, macranthoidin A, Hederagenin, cauloside C, Akebia saponin D, and α-Hederin as oleanane-type saponins (Liu et al., 2013). The triterpenoid saponins in *LM* exhibit significant anticancer, anti-solid and nonsolid tumor, antiviral, free radical scavenging, antioxidant, and liver protection activities both *in vitro* and *in vivo* and thus have substantial developmental potential (Fan et al., 2018; Chen et al., 2023, Chen et al., 2020). In this study, 134 terpenoids were tentatively identified by metabolite profiling using UHPLC-MS/MS. Our results reflected the rapid accumulation of most triterpenoids in *LM* at stages F1, F2, and F3, with the peak in stage F4, followed by a reduced level. Terpenoids, comprising more than 556 odor compounds, constitute the most diverse class of plant floral volatiles (Dötterl and Gershenzon, 2023). The main types of plant floral compounds are monoterpenes and sesquiterpenes, which play important roles in the presentation of different plant aromas, such as in herbaceous plants including *Lilium* sp (Yang et al., 2022), *Hedychium* (Ke et al., 2021), and woody plants including *Rosa damescena* (Shi and Zhang, 2022), *Osmanthus fragrans* L (Sheng et al., 2021), *Jasminum sambac (L.)* Ait (Wang et al., 2022d). Monoterpenoid and sesquiterpenoid components with differential accumulation at different flowering stages were identified in the two varieties, mainly in the mid-to-late stages of flower growth characterized by flowers movements and opening. We speculated that this accumulation pattern is the possibly associated with variable aroma during development. Although triterpenoid saponins in different plants share a common MVA and MEP skeleton synthesis pathways, the post-modification processes from 2,3-oxidized squalene to the triterpenoid saponin formation involve different patterns, resulting in multiple complex structures. Multiple key enzymes derived from the MEP and MVA pathways, which included synthases, intermediates, and transferases enzymes which involved in the synthesis and metabolism pathways of terpenoids were identified using RNA-seq data. These genes exhibit distinct tissue-specific expression patterns during the flower development cycle. TPS genes play crucial roles in plant terpene metabolism by modifying the structural types of various terpenes. The catalytic formation of terpenoids by TPS also requires further modifications by glycosyltransferases, cytochrome P450 monooxygenases, methyltransferases, acetyltransferases and short-chain dehydrogenases to produce a diverse variety of terpenoids (Cheng et al., 2020; Sawai and Saito, 2011). Most terpenoid-related *CYP*s are considered members of the *CYP*71 subfamily and many are enzymes that catalyze terpenoid oxidation. *UGT* 71, 73, 74, 85, and 91 clans have been reported participate in saponin biosynthesis (Rahimi et al., 2019), among which the *UGT*73 clan is considered as candidate gene crucial for the biosynthesis of oleanane-type triterpenoid saponin owing to the catalytic activity for the production of α - hederin from cauloside A (Yin et al., 2023). This function forms the evolutionary basis for the diversity of terpene skeletons. In this study, 15 *TPS* genes, 9 *CYP450*s and 2 *UGT*s genes involved in skeleton modification were identified in the WGCNA gene module. These genes constitute the core gene set and play an irreplaceable role in the *LM* terpenoid biosynthesis and metabolic pathways. Several studies have confirmed that members of multiple TF families, including the *MYB*, *bHLH*, *WRKY*, *ERF/AP2*, *bZIP*, and *ARF* TF family proteins, participate as regulatory factors in terpenoid biosynthesis (Huang et al., 2019; Xu et al., 2022; Man et al., 2023). In some species, *MYC*2 TFs belonging to the *bHLH* family positively regulate the gene expressions by directly binding to G-box-like motifs in the *TPS* and *CYP* promoters. In Panax notoginseng, ginsenoside biosynthesis is regulated by TFs, including *WRKY*, *AP2*, *DELLA*, *ERF3*, and *MYC2*, through the JA pathway via *PgLOX*6 (Rahimi et al., 2016). In this study, several TFs, including 12 *bHLH*s, 8 *MYB*s, 4 *AP2/ERF* family members and others, which are co-expressed with terpenoid pathway genes and play important regulatory roles, were overexpressed in the WGCNA module, suggesting that these candidate TFs may participate in the biosynthesis of terpenoids in *LM*.

### 4.4 Regulatory network involved in the specific accumulation of hormone signaling-TF-gene-secondary metabolites

**Figure 8:**
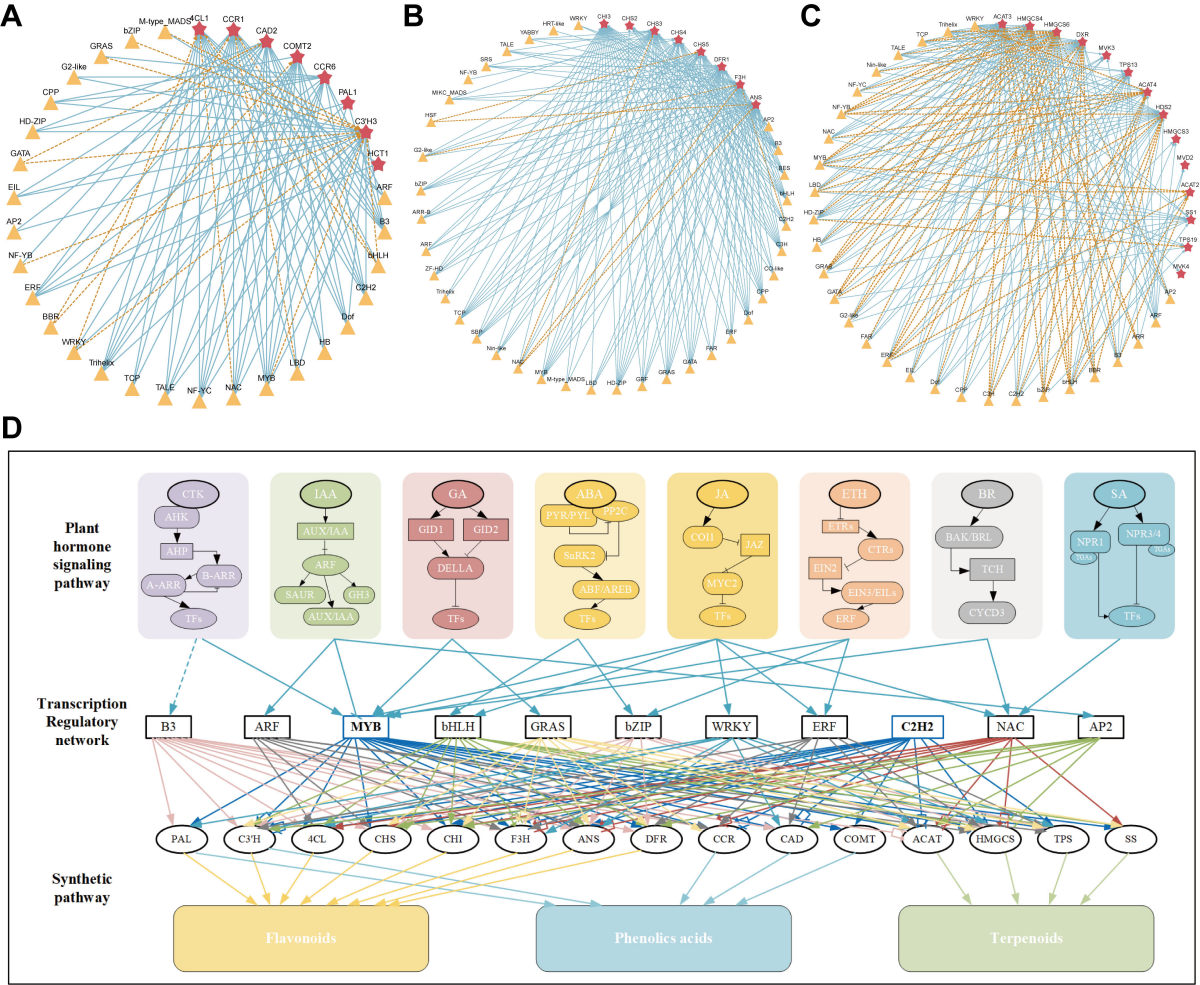
Transcriptional regulatory network of active ingredients in *LM*. The red star represent key structural genes, and the yellow triangle represent key TFs. The connecting lines with arrows and dashed line represent positive and negative regulation, respectively. Different colored lines were used to distinguish the regulation of structural genes by different TF. **(A)** Regulatory network of phenolic acids biosynthesis in *LM*. **(B)** Regulatory network of flavonoids biosynthesis in *LM*. **(C)** Regulatory network of terpenoids biosynthesis in *LM*. **(D)** The network diagram of several hormones involved in the transcription factor regulation of downstream active component structural genes.

Previously, several active ingredients including flavoids, terpenoids, alkaloids, saponins, and phenolic acids, were isolated and identified from medicinal plants owing to their crucial roles in the development of drugs and the promotion of human health (Wang et al., 2023). Numerous reports reflected that the transcriptional regulation of the biosynthesis of active ingredients in medicinal plants involves complex and multilayered mechanisms, enhancing its importance in molecular pharmacology research (Zheng et al., 2023; Shi et al., 2024). A complex network of upstream transcriptional regulators comprises different plant hormones that are considered key signals for plant growth and developmental processes, including root elongation, tillering, flowering, and fruit ripening (Mazzoni-Putman et al., 2021). As macroscopic upstream regulatory factors, plant hormones usually indirectly regulate the transcription of transcription factors such as *MYB*s, *bHLH*s, and *ERF*s through specific signaling pathways (Tong et al., 2024; Wu et al., 2021). Transcription factors regulate the activation or inhibition of downstream target genes, thereby regulating the expression of multiple downstream target genes with multiple functions. These functional TFs belong to multiple families and are induced by various hormones, which have been shown to regulate the biosynthesis of active ingredients. For instance, both auxin and ethylene can increase the transcription of *AtCHS, AtCHI*, and *AtFLS* during seed development in Arabidopsis, thereby increasing the anthocyanins and flavonols contents. However, *AtF3’H* is significantly increased by auxin only (Lewis et al., 2011). Extensive research has shown that JA plays an important role in regulation of saponin accumulation. The TF *PgbHLH28* promotes saponin content by inducing the expression of *PgHMGR2 and PgDXS2*, and mediates JA signaling in *Platycodon grandiflorus* (Zhang et al., 2024). The expression of *SmMYB111* is significantly downregulated by MeJA, GA, or SA treatment (Li et al., 2018). Many hormone-related genes involved in the biosynthesis of *LM* active ingredients were identified using transcriptional data analysis. KEGG analysis also revealed that plant hormone signal transduction pathways were significantly enriched in multiple comparison groups, suggesting that plant signal transduction potentially plays an important role in the flower development of *L. macranthoides*, simultaneously affecting the accumulation of active ingredients. Through a combined analysis of transcription and metabolism, we further identified hormone-related genes and key transcription factors involved in the regulatory network associated with active ingredients, thus forming a bridge between hormone signaling TF structural genes. A network diagram of several hormones involved in the transcription factor regulation of downstream active component structural genes is shown in **Figure 8**.

## 5 Conclusions

Overall, our investigation in this study firstly performed an integrative analysis of the transcriptome and metabolome of seven stages across wild-type and xianglei-type *Lonicera macranthoides* to explore the related genes and metabolites associated with flavonoid, phenolic acid, and terpenoid biosynthesis and accumulation patterns. During the late stage of flower development, the accumulation of these active ingredients significantly differed between the two tested *LM* varieties. Moreover, 36 hub-gene and major transcription factors *NAC*, *MYB*, *WRKY*, and *bHLH* were identified via comprehensive analysis. Furthermore, our analysis revealed that the phytohormone signaling pathway crucially regulates the accumulation of active ingredients during flower development in *LM*. Overall, this study focused on the correlation between phenotype and the active components and mapped the changes in the regulatory network of bioactive compounds during the development of wild-type and xianglei-type *LM*s to elucidate the biological regulatory mechanisms associated with the active components of *LM*s. The results provide some insights into the breeding direction of floral medicinal plants. Further characterization of the effective active substances in these medicinal plants is needed focusing on the transcriptional regulation mediated by environmental factors, transcription factors, and plant hormones, as well as their interactions. Combined analysis of transcriptome and metabolome is an effective means to study the regulatory mechanism of plant metabolite synthesis, but it still has limitations. Firstly, the dynamic changes of gene expression and metabolites may not be synchronized, such as gene expression earlier than metabolites; Secondly, the candidate genes and metabolites selected by omics joint analysis mostly depend on bioinformatics prediction, and lack experimental validation, such as gene knockout, overexpression, quantitative metabolite tracking, etc., which will be more helpful for the subsequent specific functional verification and mechanism discussion. Through dynamic sampling design, multi-omics integration, spatial technology application and synthetic biology verification, the understanding of the regulatory mechanism of active components can be significantly improved.

## Data Availability

The datasets generated and analyzed during the current study are available in the NCBI Sequence Read Archive (SRA) database. The raw sequence data are available at NCBI, with Bioproject accession number PRJNA1040459.
