# Supplementary material for: Accumulation differences of high-value ingredients in different phenotype Lonicera macranthoides: insights from integrative metabolome and transcriptome analyses
Source: Front Plant Sci. 2025 Mar 4;16:1533263. doi: 10.3389/fpls.2025.1533263 (PMC11913843; doi:10.3389/fpls.2025.1533263)
Supplement: Supplementary file 1 [file Presentation1.zip › supplementary files/supplement figure.docx]

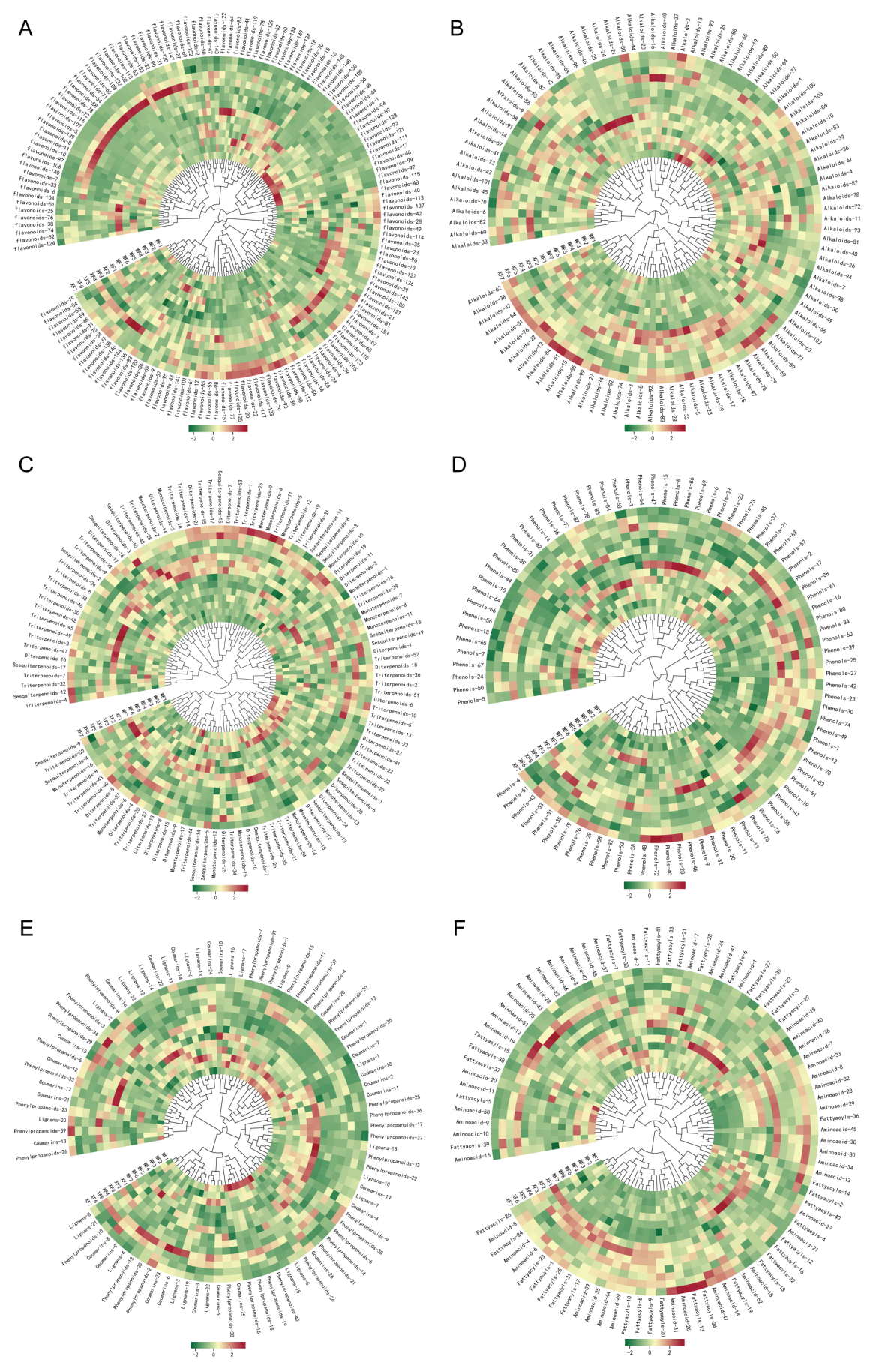


Figure S1. (A) Heat map of the contents of flavonoids of WT and XL at different stages of development, (B) Heat map of the contents of Alkaloidsof WT and XL at different stages of development, (C) Heat map of the contents of Terpenoids of WT and XL at different stages of development, (D) Heat map of the contents of Phenols of WT and XL at different stages of development, (E) Heat map of the contents of Phenylpropanoids of WT and XL at different stages of development. (F) Heat map of the contents of Phytohormone, Benzene and derivatives, Carbohydrates, Organicoxygen and Organicnitrogen compounds, Steroids, Fatty acyls of WT and XL at different stages of development. (G) Heat map of the contents of Amino acids and derivatives, Nucleotide and derivates, Organicacids and derivatives, Carboxylicacids and derivatives of WT and XL at different stages of development. WF1-WF7 represent the seven developmental stages of ‘Wild’ type LM, and XF1-XF7 represent the seven developmental stages of ‘XiangLei’ type LM.


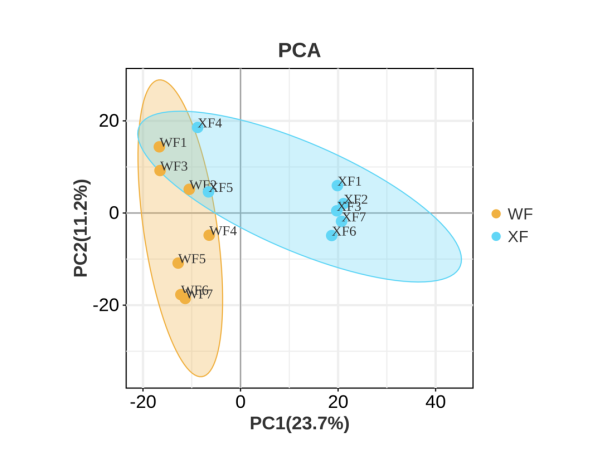

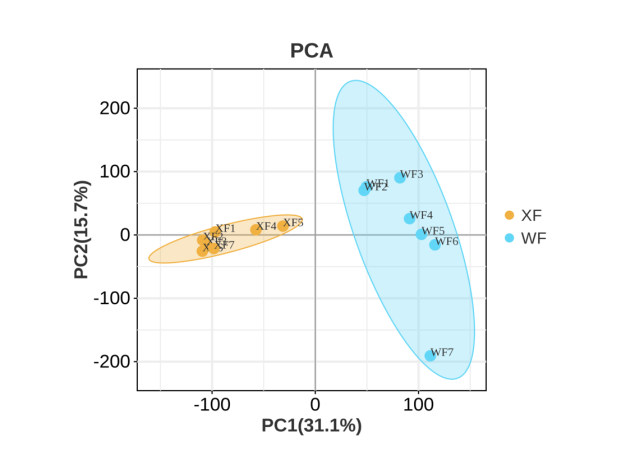


Figure S2. The PCA score plot of samples in metabolomic(A) and transcriptomic(B) profile of WT and XL.


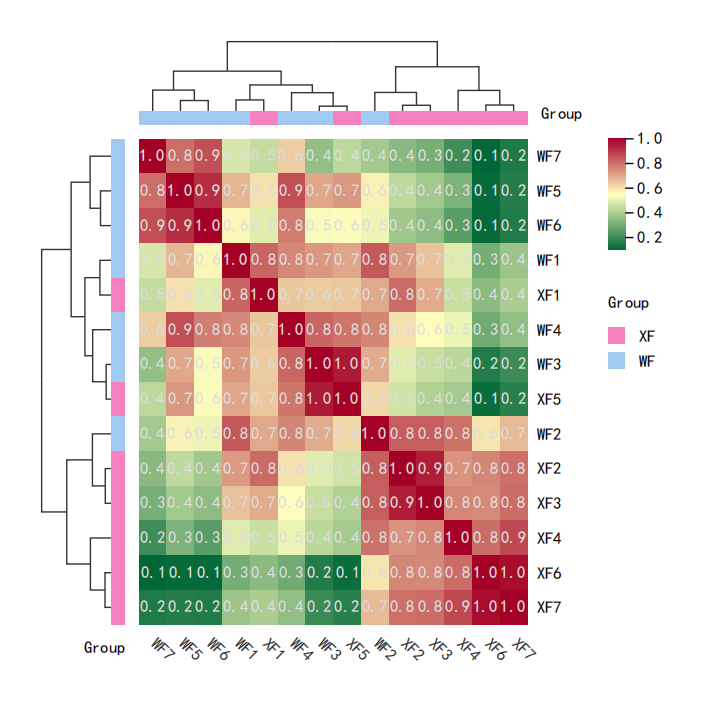


Figure S3. The correlation values of samples in the seven stages of WT and XL.


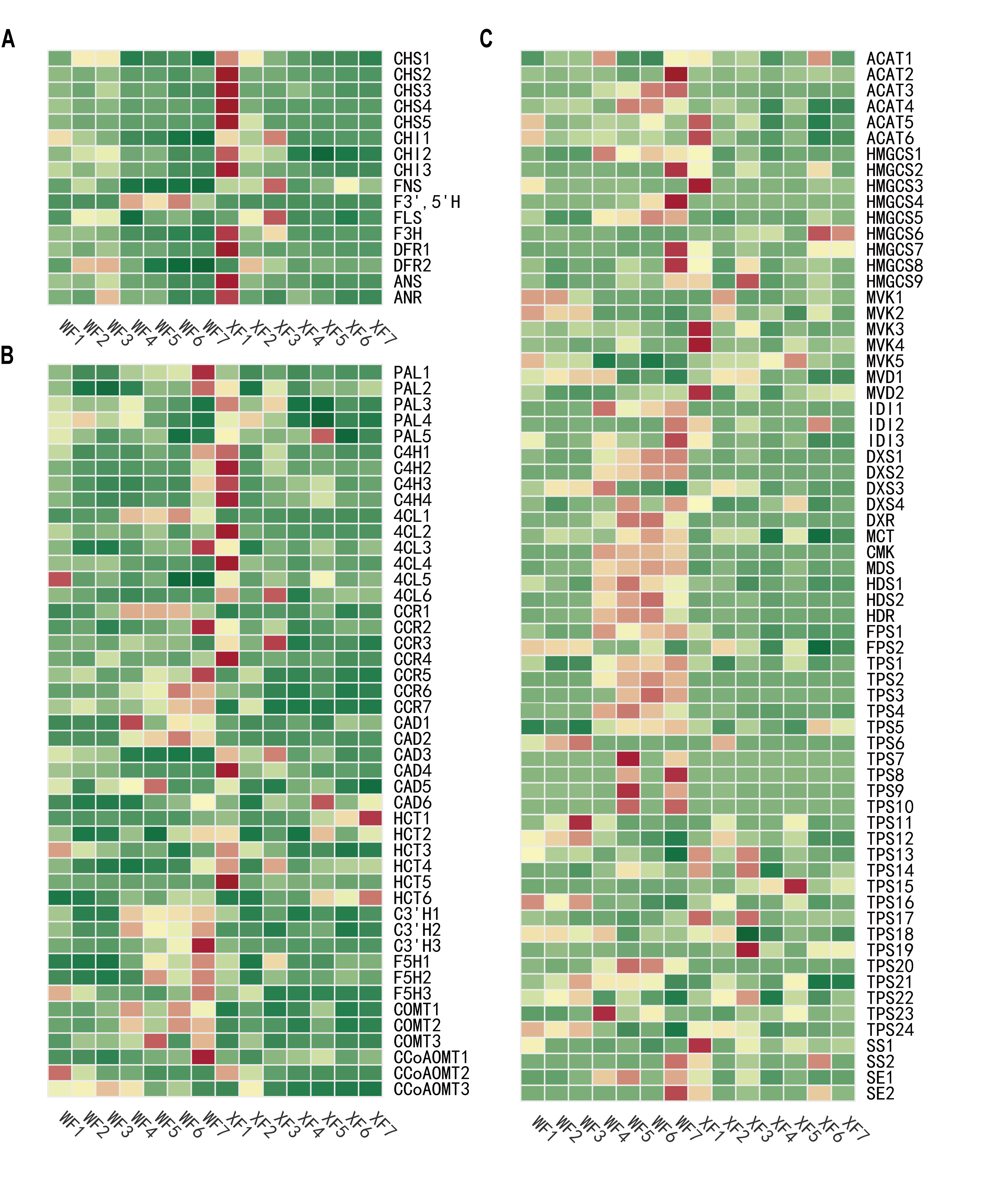


Figure S4. (A) Expression levels of structual genes involved in flavonoids biosynthesis. (B) Expression levels of structual genes involved in phenolic acid biosynthesis. (C) Expression levels of structual genes involved in terpenoid biosynthesis.
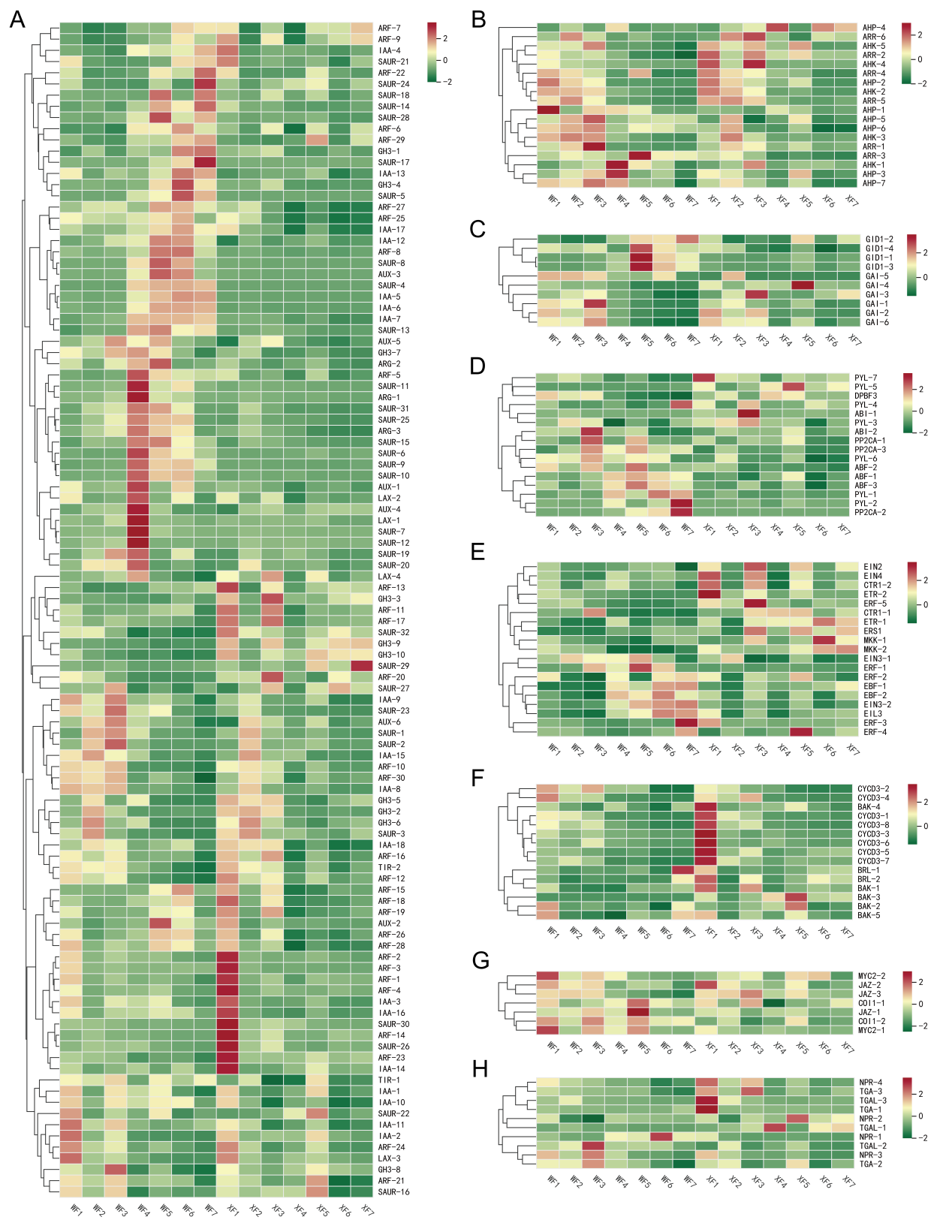


Figure S5. (A) Expression levels of DEGs in IAA signaling. (B) Expression levels of DEGs in CTK signaling. (C) Expression levels of DEGs in GA signaling. (D) Expression levels of DEGs in ABA signaling. (E) Expression levels of DEGs in ETH signaling. (F) Expression levels of DEGs in BR signaling. (G) Expression levels of DEGs in JA signaling. (H) Expression levels of DEGs in SA signaling.


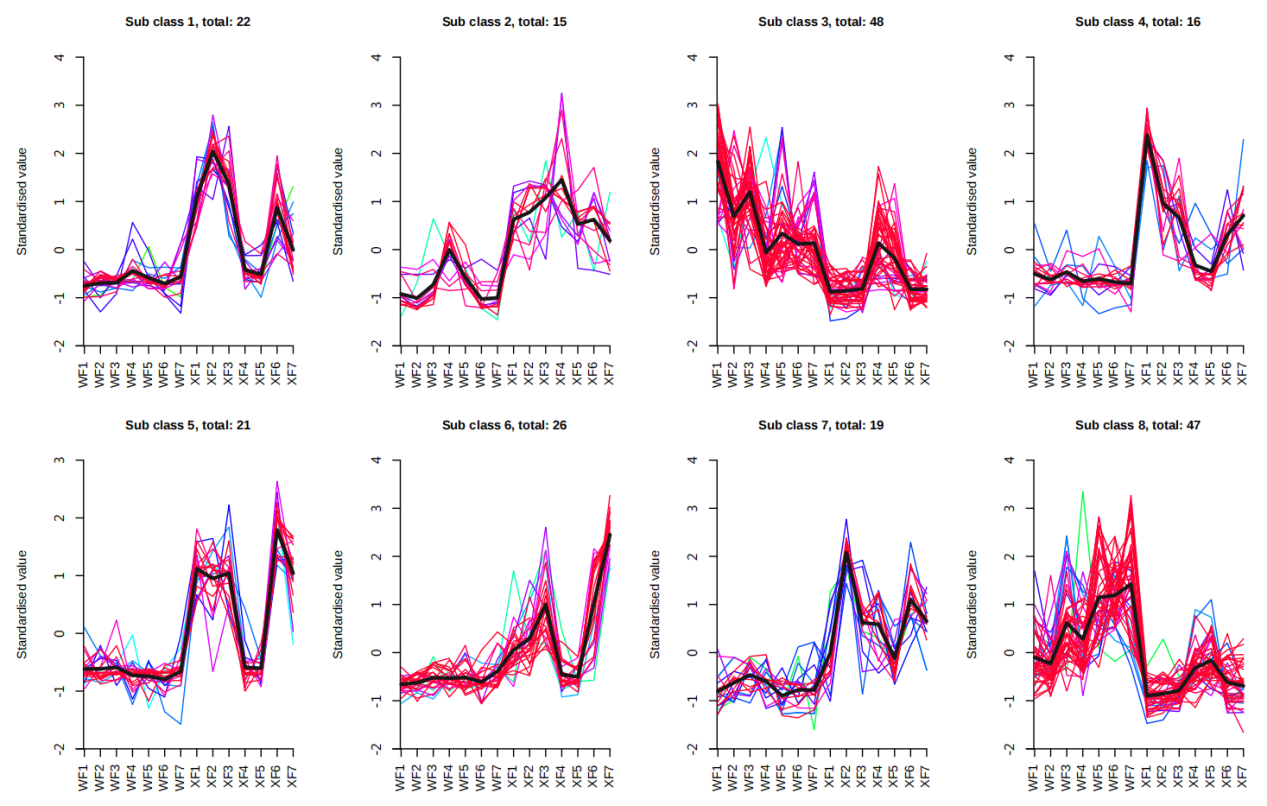


Figure S6. Mfuzz clustering on the basis of 647 significant DAMs content.


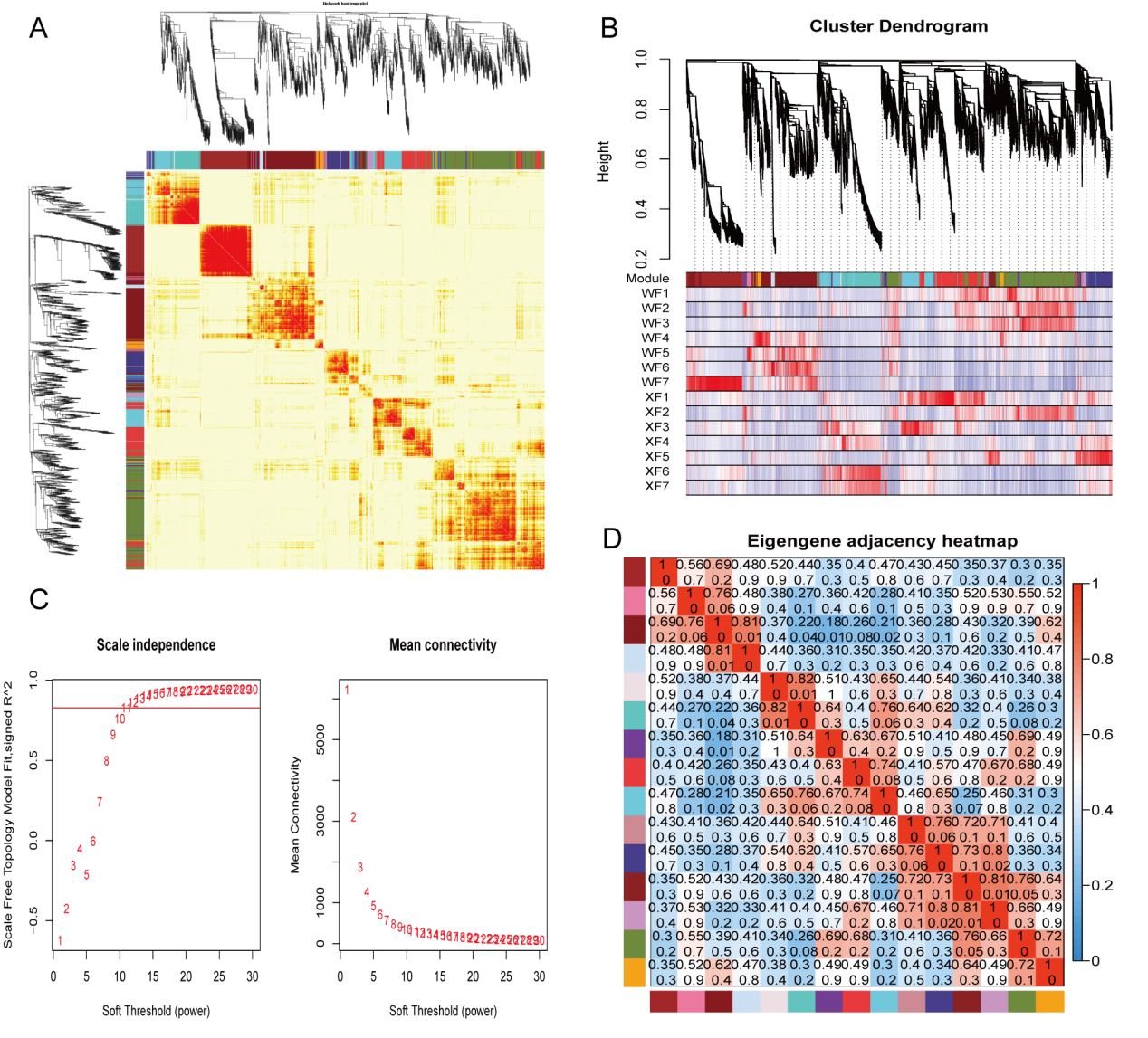


FigureS7. Gene co-expression network analysis. A.The network Heatmap B.The gene dendrogram with flower stages. C.The softpower plot D.the hubGene Heatmap.


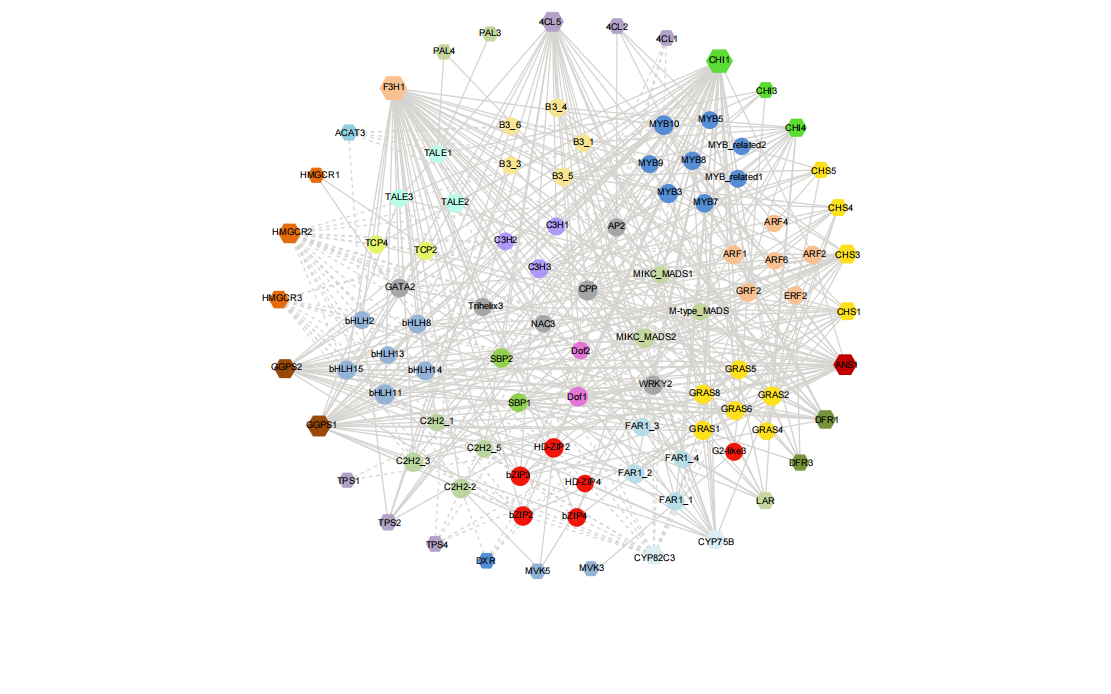


Figure S8.The regulatory network of TFs and key structural genes.
